# Activity-dependent regulation of MHC class I expression in the developing primary visual cortex of the common marmoset monkey

**DOI:** 10.1186/1744-9081-7-1

**Published:** 2011-01-04

**Authors:** Adema Ribic, Gabriele Flügge, Christina Schlumbohm, Kerstin Mätz-Rensing, Lutz Walter, Eberhard Fuchs

**Affiliations:** 1 Clinical Neurobiology Laboratory, German Primate Center/Leibniz Institute for Primate Research, Kellnerweg 4, Göttingen 37077, Germany; 2 Primate Genetics Laboratory, German Primate Center/Leibniz Institute for Primate Research, Kellnerweg 4, Göttingen 37077, Germany; 3 Pathology Unit, German Primate Center/Leibniz Institute for Primate Research, Kellnerweg 4, Göttingen 37077, Germany; 4 Department of Neurology, Medical School, University of Göttingen, Göttingen, Germany; 5 DFG Research Center Molecular Physiology of the Brain (CMPB), University of Göttingen, Göttingen, Germany; 6 Department of Molecular Biophysics and Biochemistry, Yale University, 333 Cedar Street, New Haven, CT 06520-8024, USA

## Abstract

**Background:**

Several recent studies have highlighted the important role of immunity-related molecules in synaptic plasticity processes in the developing and adult mammalian brains. It has been suggested that neuronal MHCI (major histocompatibility complex class I) genes play a role in the refinement and pruning of synapses in the developing visual system. As a fast evolutionary rate may generate distinct properties of molecules in different mammalian species, we studied the expression of MHCI molecules in a nonhuman primate, the common marmoset monkey (*Callithrix jacchus*).

**Methods and results:**

Analysis of expression levels of MHCI molecules in the developing visual cortex of the common marmoset monkeys revealed a distinct spatio-temporal pattern. High levels of expression were detected very early in postnatal development, at a stage when synaptogenesis takes place and ocular dominance columns are formed. To determine whether the expression of MHCI molecules is regulated by retinal activity, animals were subjected to monocular enucleation. Levels of MHCI heavy chain subunit transcripts in the visual cortex were found to be elevated in response to monocular enucleation. Furthermore, MHCI heavy chain immunoreactivity revealed a banded pattern in layer IV of the visual cortex in enucleated animals, which was not observed in control animals. This pattern of immunoreactivity indicated that higher expression levels were associated with retinal activity coming from the intact eye.

**Conclusions:**

These data demonstrate that, in the nonhuman primate brain, expression of MHCI molecules is regulated by neuronal activity. Moreover, this study extends previous findings by suggesting a role for neuronal MHCI molecules during synaptogenesis in the visual cortex.

## Background

The major histocompatibility complex (MHC) is a dense cluster of genes found in all jawed vertebrates that encodes a great number of proteins involved in immune responses [1,2]. A group of these genes, MHC class I, codes for transmembrane glycoproteins responsible for presentation of antigenic peptides to cytotoxic CD8+ T lymphocytes [3]. MHC class I (MHCI) proteins are typically heterotrimers composed of a polymorphic transmembrane heavy chain (HC), a noncovalently attached ß-2-microglobulin subunit, and a short peptide comprising 8-15 amino acids that is derived from self or foreign antigens [4,5]. In this form, MHCI modulates immune response by interacting *in trans *with a large number of immune receptors [6]. Additionally, MHCI heavy chains can be detected under certain conditions on the cell surface without ß-2-microglobulin and peptide [6]. It is thought that in this "free heavy chain" form, MHCI molecules may interact *in cis *with certain receptors and thereby regulate their trafficking [6-8]. Virtually all nucleated cells express MHCI proteins, usually in their heterotrimeric form; however, their expression on neurons was always debated [9]. Despite the controversy, neuronal expression of MHCI under certain conditions has been reported [10,11] and recent findings on involvement of neuronal MHCI in brain development and synaptic plasticity were of great surprise [12-15]. These and other studies begun to unravel the previously unknown and complicated mechanisms of interactions between the central nervous and the immune system that may have great clinical implications [16].

Although MHCI genes are well conserved among mammals, there are a number of differences in the organization, structure, and function of these genes between rodents and primates, including humans [17]. The first study on the function of MHCI molecules in the central nervous system (CNS) proposed a role for them in the removal of excess synapses in the developing visual system [15]. The visual system displays two forms of plasticity: visual input-driven, activity-dependent plasticity and activity-independent plasticity [18]. The development of the main structures of the visual system, the thalamus and the primary visual cortex (V1), are at least at certain developmental stages dependent on the activity coming from the retina [19,20]. The thalamic dorsolateral geniculate nucleus (LGN) is the first relay structure of visual input and is organized into segregated, eye-specific neuronal layers that form upon stimulation by early spontaneous activity of retinal ganglion cells [19]. Neurons of the LGN send their projections to V1 where their activity is needed in proper development of eye-specific populations of neurons assembled in the ocular dominance columns (ODCs; [18]). Blockade of activity of one eye during development of the visual circuits while leaving the other one intact (monocular deprivation) perturbs the segregation of the LGN neurons into eye-specific layers [19,21]. In V1, as a consequence of visual deprivation, the population of neurons responsive to the intact eye increases [18,22]. Monocular deprivation by means of tetrodotoxin-induced block of retinal activity downregulates MHCI expression levels in the LGN [15]. Furthermore, it was reported that MHCI molecules are indispensable for developmental refinement in the LGN, i.e. for removal of excess synapses during normal development [12,23]. Asides from differences in the structure and function of MHCI genes, primates and rodents differ significantly in terms of structure of their visual systems [24]. To gain an insight into the role of MHCI molecules in the nonhuman primate brain, we investigated the spatiotemporal pattern of expression of MHCI genes in the primary visual cortex of the common marmoset monkey. Our data confirmed that MHCI molecules are expressed in the developing visual cortex of the marmoset monkey and that their levels are regulated by neuronal activity. However, the temporal pattern of their expression during development of the V1 and their expression levels upon monocular enucleation indicated that MHCI molecules investigated here may not be involved in synapse elimination, but rather in synaptogenesis. These results not only validate the potentially important role of neuronal MHCI molecules in visual cortex development, but also point to interesting interspecies differences in their distribution, and potentially their function.

## Methods

### Animals

Thirty-six common male marmoset monkeys (*Callithrix jacchus*) were investigated. The animals were obtained from the breeding colony at the German Primate Center (Göttingen, Germany). All investigations and the experiments were performed to the highest ethical standards according to the relevant local, national and international regulations concerning the use of animals. All research projects were carried out only with authorization from the relevant ethics committees. We employed the minimum number of animals required to obtain consistent data and to avoid animal suffering. For the termination of non-human primates, the legal requirements, guidelines and recommendations set by the national authorities were followed strictly. The use of animals including non-human primates in research in Germany is based on the "Tierschutzgesetz" (Animal Protection Law) of the 25^th ^of May, 1998 (BGBl.I Nr.30 v.29.5.1998, S. 1105) and the EU guidelines 86/609/EEG. The proposals for research conducted in this study are approved by the local Government and a specially appointed Ethical Review Committee (Niedersächsisches Landesamt für Verbraucherschutz und Lebensmittelsicherheit, permit numbers 33.11.425-04-003/08 and 33.11.425-04-026/07).

For expression studies, animals of the following ages were used [25,26]: postnatal days 1 and 7, and postnatal months 1, 3, 5, 7, 12, and 21 (N = 3 per age). For monocular enucleation (ME), the left eyes of one month-old marmoset monkeys (N = 6; approximate body weight 75 g) were surgically enucleated under general anesthesia. As anesthesia, the animals received 0.1 ml of a premix containing 4 mg/ml alphaxalon and 1.33 mg/ml alphadolon (Saffan^®^; Schering-Plough Animal Health, Welwyn Garden City, UK), 0.37 mg/ml diazepam, and 0.015 mg/ml glycopyrroniumbromid (Robinul^®^; Riemser, Germany). Enucleation was carried out as described for pet animals [27] and the eyehole was filled with Gelastypt^® ^sponge (Sanofi-Aventis, Frankfurt, Germany) as soon as arterial bleeding was no longer visible. The wound was closed with an intracutane suture of vicryl 6-0 (V302H; Ethicon, Norderstedt, Germany). Directly after surgery (day 0) and at days 3 and 5, all animals received an antibiotic (amoxicillin-trihydrate; Duphamox LA^®^, Fort Dodge, Würselen, Germany). ME animals and six age-matched controls were sacrificed at five months of age [28].

### Brain sections

For *in situ *hybridization, brains were immediately removed from terminally anesthetized animals [overdose of ketamine (50 mg/ml), xylazine (10 mg/ml), and atropine (0.1 mg/ml)] and the whole visual cortices were quickly dissected (area determined according to [29,30]), embedded in Tissue Tek (Sakura Finetek, Heppenheim, Germany), flash frozen in liquid nitrogen, and stored at -80°C. Frozen brains were sectioned using a cryostat (Leica CM3050, Bensheim, Germany) and coronal sections (10 μm) of whole visual cortices were thaw-mounted on adhesive silane-coated slides (Histobond, Marienfeld, Laboratory Glassware, Lauda-Königshofen, Germany). For immunohistochemistry, marmosets that were terminally anesthetized (see above), were perfused transcardially with 0.9% saline, followed by 200 ml of fixative containing 4% paraformaldehyde in 0.1 M sodium-phosphate buffer (pH 7.2) for 15 min. The heads were postfixed in the same fixative, and brains were carefully removed from the skulls on the following day. After cryoprotection in 0.1 M phosphate-buffered saline (PBS; 0.1 mM phosphate buffer, 0.9% NaCl, pH 7.2) containing 30% sucrose, serial coronal sections of the entire visual cortices (thickness: 40 μm for expression studies and 60 μm for ME animals and controls) were obtained using a cryostat.

### PCR cloning of MHCI transcripts

The isolation of the common marmoset MHC class I heavy chain cDNA sequences was carried out using reverse transcriptase polymerase chain reaction (RT-PCR). One microgram of total brain RNA was reverse transcribed using the oligo (dT) primer and 400 U of reverse transcriptase (Promega, Mannheim, Germany). An aliquot of this reaction was used as a template in a PCR containing primers designed with the Primer3 software [31]. Primers were devised to amplify full-length marmoset MHC class I heavy chain transcripts (accession number: U59637). Primer sequences also included BamHI restriction sites and were as follows: forward 5'-CACGGATCCCACTTTACAAGCCGTGAGAGAC-3', reverse 5'-CACGGATCCCTCCTGTTGCTCTCGGGGGCCTTG-3'. Caja-G*1 (accession number: U59637) was obtained and cloned into the pDrive vector (Qiagen).

### *In situ *hybridization

Cryosections (10 μm) of visual cortices were dried at RT for 20 min, fixed in 4% buffered paraformaldehyde (PFA, pH 7.2), rinsed in PBS, acetylated (0.1 M triethanolamine, 0.25% acetic acid anhydride), washed in PBS, and dehydrated through a graded ethanol series. Caja-G plasmid DNA was linearized and riboprobes were synthesized using T7 and SP6 RNA polymerases (Promega, Madison, WI, USA) for the antisense and sense probe, respectively, in the presence of 9.25 MBq of ^33^P-UTP (Hartmann Analytic GmbH, Braunschweig, Germany; specific activity, 3,000 Ci/mmol) for 1 h at 37°C. Probes were purified using Microspin S-400 HR columns (Amersham Pharmacia, Freiburg, Germany) and hybridization buffer (50% deionized formamide, 10% dextran sulphate, 0.3 M NaCl, 1 mM EDTA, 10 mM Tris-HCl, pH 8.0, 500 μg/ml tRNA, 0.1 M dithiothreitol, and 1 × Denhardt's solution) was added to yield a final probe activity of 5 × 10^4 ^cpm. The hybridization mixture containing the probe was denatured at 70°C for 10 min, cooled to 55°C, and pipetted directly onto sections (80 μl/section). Hybridization was performed for 18 h at 68°C. Sections were subsequently washed in 4 × SSC (0.6 M NaCl, 0.06 M citric acid), 2 × SSC, and 0.5 × SSC for 10 min each at 37°C. After incubation (1 h at 75°C) in 0.2 × SSC, sections were washed twice in 0.1 × SSC, once at 37°C and again at RT, for 10 min each. Finally, sections were dehydrated through graded alcohols, air dried, and exposed to Bio-Max MR film (Amersham Pharmacia) for three days at 4°C. Films were developed and fixed with GBX (Kodak, Rochester, NJ, USA).

### Quantitative *in situ *hybridization

After *in situ *hybridization, sections were coated with photoemulsion (Kodak NBT2, Rochester, NJ, USA) at 42°C, dried for 90 min at RT, and stored for seven weeks at 4°C in a lightproof container. Exposed slides were developed at 15°C for 5 min (Kodak developer D-19), rinsed twice briefly in H_2_O, and fixed for 5 min at RT (fixer, Kodak Polymax). Sections were counterstained either with 0.05% toluidine blue in 0.1% disodium tetraborate (Sigma) for expression studies or with methyl green (Sigma) for monocular enucleation studies, cleared in xylol, and coverslipped using mounting medium (Eukitt, Kindler, Freiburg, Germany). Hybridized sections were visualized with a 40× objective (NA = 1.4; Zeiss) under a light microscope (Axioscope, Zeiss) and silver grain quantification was performed on a cell-by-cell basis using ImageJ (U.S. National Institutes of Health, Bethesda, Maryland, USA). Images were obtained from layer IV neurons and, for each area within the primary visual cortex (demarcated according to [29,30]) two images were acquired, i.e., one using a green filter to eliminate background staining from methyl green and one using white light to later precisely localize neuronal nuclei. A circular counting mask of 15 μm in diameter was used to delineate the region of interest and was placed over neuronal nuclei during counting. Relative optical density (ROD) threshold intensities were optimized to detect exposed silver grains exclusively. The silver grain density within the region of interest was measured. Grain density was compared between layer IV neurons in primary visual cortices of six animals (three slides per animal, minimum 300 neurons per animal were counted) using Student's t test (GraphPad Prism version 4 for Windows, GraphPad Software, San Diego, CA, USA).

### Quantitative RT-PCR

To isolate RNA for RT-PCR, brains were immediately removed from terminally anesthetized animals (see above) and whole occipital lobes containing visual cortices were quickly dissected. Total RNA was isolated from both hemispheres of dissected tissue samples from animals at the age of five months (N = 3 controls and N = 3 unilaterally enucleated animals) using the QIAGEN RNeasy kit (Qiagen, Hilden, Germany) according to the manufacturer's instructions. As the cortices had to be separated during RNA isolation due to their size, both left and right hemispheres of all animals were treated as independent samples. The integrity and quantity of purified RNA was assessed by spectrophotometry. Complementary DNA (cDNA) was synthesized from mRNA transcripts using oligo (dT)_12-18 _primers and Superscript II reverse transcriptase (Invitrogen, Karlsruhe, Germany), according to the manufacturer's instructions. The Primer3 software v2.0 [31] was used to design gene-specific primers, with amplicons ranging from 50 to 150 bp in length. The primers used for the detection of MHC class I heavy chain transcripts were: forward 5'-GTGATGTGGAGGAAGAACAGC-3', reverse 5'-CACTTTACAAGCCGTGAGAGA-3' (CajaG*01, accession number U59637). Primers for the detection of GFAP were: forward 5'-AAACGAGTCCCTGGAGAG-3', reverse 5'-TCCTGGTACTCCTGCAAGT-3' (marmoset GFAP, Ensembl transcript number ENSCJAT00000024380). Primers for the detection of c-Fos were: forward 5'-CGAAGGGAAAGGAATAAGAT-3', reverse 5'-GCAGACTTCTCATCTTCCAG-3' (marmoset c-Fos, Ensembl transcript number ENSCJAT00000040535). Primers for the detection of β-actin were: forward 5'-CATCCGCAAAGACCTGTATG-3', reverse 5'-GGAGCAATGACCTTGATCTTC-3' (marmoset ß-actin, accession number DD279463). A quantitative analysis of gene expression was performed using the 7500 Real-time PCR apparatus (Applied Biosystems, Darmstadt, Germany) in combination with Quantitect SYBR green technology (Qiagen). The light cycler was programmed to the following conditions: an initial PCR activation step of 10 min at 95°C, followed by 40 cycling steps (denaturation for 15 s at 95°C, annealing for 30 s at 55°C, and elongation for 60 s at 72°C). Details of the quantitative real-time PCR were described previously [32]. Dissociation curves were generated for all PCR products to confirm that SYBR green emission was detected from a single PCR product [33]. All products were run on 2.5% agarose gels to confirm a single product (Additional file 1). The Caja-G PCR product was additionally sequenced using the BigDye Terminator Sequencing Kit (Applied Biosystems) and forward primer in order to confirm its identity (Additional File 2). The relative abundance of the MHCI-HC, c-Fos and GFAP mRNA transcripts was calculated relative to the mRNA levels of the internal reference gene β-actin and was compared between both left and right cortices of control and enucleated animals using Student's *t*-test (GraphPad Prism version 4 for Windows).

### Immunocytochemistry for light microscopy

Coronal cryosections (40 μm for expression studies and 60 μm for ME animals) of occipital lobes were collected and washed briefly in PBS before the epitope retrieval step. Epitope retrieval was performed by incubating the sections for 20 min in 10 mM sodium citrate buffer preheated to 80°C. Sections were later brought to RT, washed in PBS, and quenched of endogenous peroxidase activity using 30 min incubation at RT in 0.5% H_2_O_2 _in distilled water. Sections were then washed in PBS, blocked for 1 h at RT (3% normal horse serum in PBS), incubated for 16 h at 4°C with mouse monoclonal TP25.99 or Q1/28 IgG [34,35] kindly provided by S. Ferrone, University of Pittsburgh, USA), 1:300 dilution in 3% normal horse serum in PBS; monoclonal mouse W6/32 [36], 1:300 dilution in 3% normal horse serum in PBS; monoclonal rabbit anti c-Fos (Cell Signalling Technologies, Beverly, MA, USA); monoclonal anti-GFAP (Sigma) 1:200 dilution in 0.01% Triton-X 100 and 3% normal horse serum in PBS; or control mouse IgG (Sigma), and washed again. For c-Fos, W6/32 and GFAP staining, the epitope retrieval step was not performed. Sections were then incubated with biotinylated horse anti-mouse IgG or donkey anti-rabbit IgG (Vector Laboratories, Burlingame, CA, USA), 1:200 dilution in 3% normal horse serum in PBS, for 1 h at RT. After washing, sections were incubated with avidin-biotin horseradish peroxidase (Vectastain Elite ABC Kit, Vector Laboratories, Burlingame, CA, USA), 1:100 dilution in 3% normal horse serum in PBS, for 1 h at RT, washed in PBS and then again in 0.05 M Tris/HCl (pH 7.2) prior to DAB detection (DAB detection with or without nickel enhancement was performed according to the manufacturer's instructions; DAB-Kit, Vector Laboratories). Sections were washed in 0.05 M Tris/HCl (pH 7.6) and again in 0.1 M PBS prior to xylol clearance, dehydration, and coverslipping with Eukitt mounting medium (Kindler). For identification of cortical layers, sections adjacent to the ones used for immunocytochemistry were stained with toluidine-blue (Sigma). Digital images of stained sections were acquired using an Axiophot II microscope (Zeiss). Final images were assembled in Corel PhotoPaint X3 and, in case of higher magnifications, were a composition of 4-5 images along the longitudinal axis of the primary visual cortex [29,30]. Contrast and luminosity were adjusted in Corel PhotoPaint X3 for images obtained from monocularly enucleated animals.

### Immunofluorescence and confocal microscopy

Antibodies used in double-labeling experiments were applied sequentially and blocking steps were performed using normal sera of the host species from which the respective secondary antibodies were derived. Cryostat sections (40 μm) of occipital lobes were rinsed in PBS before the epitope retrieval step was performed, as described above. Nonspecific antibody binding sites were blocked with 3% normal serum in PBS for 1 h at RT. Sections were then incubated with mouse monoclonal TP25.99 antibody, 1:300 in 3% normal serum in PBS, for 16 hrs at 4°C, washed, and incubated in secondary antiserum (Alexa 488-coupled goat anti-mouse, Molecular Probes, Invitrogen, Leiden, Netherlands) at a dilution of 1:500 for 4 h in a lightproof container. Sections were then washed and incubated with either rabbit anti-MAP2 antibody (1:200, Synaptic Systems, Göttingen, Germany), rabbit anti-NMDAR1 (1;200, Synaptic Systems), rabbit anti-GFAP (1:500, Synaptic Systems), or rabbit anti-vimentin antibody (1:200, Synaptic Systems) in 3% normal serum in PBS for 16 h at 4°C. For GFAP-vimentin colocalization studies, the epitope retrieval step was not performed and following primary antibodies were used: rabbit anti-vimentin (1:200, Synaptic Systems) and mouse anti-GFAP (1:200, Sigma). Sections were then washed and incubated 4 h at RT in secondary antiserum (Alexa 568-coupled goat anti-rabbit, Molecular Probes, Invitrogen) diluted 1:500 in 3% normal serum in PBS. Thereafter, sections were washed in PBS and floated/mounted on SuperFrost ^®^Plus slides (Menzel-Gläser GmbH, Braunschweig, Germany) in distilled water, allowed to dry overnight at 4°C, and coverslipped with mounting medium (Aqua-Polymount, Polysciences Inc., Warrington, PA, USA). For control sections, the same procedures were performed omitting the primary antibody.

Confocal microscopy was performed using a laser-scanning microscope (LSM 5 Pascal, Zeiss) with an argon 488 nm laser and a helium/neon 543 nm laser (in multiple-tracking mode). High magnification, single optical plane images of layer IV neurons of the primary visual cortex [29,30] or of the subcortical white matter in the occipital lobes were obtained at a resolution of 1,024 × 1,024 with an Apochromat 63× oil objective (NA = 1.4) and immersion oil (Immersol, Zeiss; refractive index = 1.518). The control sections (no primary antibodies) displayed only background fluorescence (data not shown).

### Protein extraction

Brains were immediately removed from terminally anesthetized animals (see above), and the whole visual cortices were quickly dissected. Whole visual cortex samples were homogenized with a Dounce homogenizer (tight pestle) in ice-cold homogenization buffer consisting of 50 mM Tris/HCl pH 7.4, 7.5% glycerol, 150 mM NaCl, 1 mM EDTA, 1% Triton-X 100, and complete protease inhibitor cocktail (Roche Diagnostics, Mannheim, Germany). After homogenization, the samples were centrifuged at 4,000× *g *for 20 min at 4°C. The resulting supernatant was centrifuged again until it was clear. Protein concentration was measured using the Bio-Rad DC Protein assay (Bio-Rad Laboratories, Hercules, CA, USA).

### Immunoblot analysis

Protein preparations were electrophoresed in 12.5% SDS gels under reducing conditions. Proteins were subsequently transferred to nitrocellulose membranes (Schleicher and Schuell, Dassel, Germany) via semidry electroblotting for 2 h at 1 mA/cm^2 ^in transfer buffer containing 25 mM Tris-base, 150 mM glycine, and 10% (v/v) methanol. After the transfer, the blotted membranes were blocked with 5% (w/v) milk powder and 0.1% Tween-20 in PBS for 1 h at RT and were then incubated with either monoclonal TP25.99 (1:1,000) or monoclonal anti-SNAP-25 (1:1,000, Synaptic Systems) antibodies overnight at 4°C. After washing three times for 5 min in PBS/0.1% Tween-20, blots were incubated for 1 h at RT with horseradish peroxidase-coupled goat anti-mouse IgG (1:4,000, Santa Cruz Biotechnology, Santa Cruz, USA). Prior to visualization, blots were washed in PBS/0.1% Tween-20 (3 × 5 min) and once more in PBS. Signals were visualized using SuperSignal West Dura enhanced luminescence substrate (Pierce Biotechnology, Rockford, IL, USA). Membranes were subsequently stripped in a mixture of β-mercaptoethanol and SDS in PBS and incubated with monoclonal anti-β-actin antibody (1:4000, Sigma). For controls, the same procedures were performed using the mouse IgG (Sigma) instead of a primary antibody. Control IgG yielded no signals in the Western blot (data not shown). For quantification, blots were visualized with MCID Basic software (Imaging Research Inc., St. Catherines, Ontario, Canada) so that nonsaturating bands were obtained. Quantification was performed using the gel analysis plug-in of ImageJ (U.S. National Institutes of Health, Bethesda, Maryland, USA). Values obtained for MHCI and SNAP-25 were normalized to ß-actin values.

### Image analysis of sections from monocularly enucleated animals

Digital images of immunostained tissue sections were acquired using an Axiophot II microscope (Zeiss). Images were taken from the V1 areas of right visual cortices. Variations in MHCI immunoreactivity through layer IV were measured as described previously [37]. Briefly, images were normalized and black-white inverted using ImageJ (U.S. National Institutes of Health, Bethesda, MD, USA) and regions within layer IV that encompassed the thickness of the entire layer IV (2 mm in length and approximately 1 mm in thickness) were defined as the regions of interest. For identification of cortical layers, sections adjacent to the ones used for immunocytochemistry were stained with toluidine-blue (Sigma). The density profile of layer IV in the primary visual cortex (region demarcated according to [29,30]) was obtained using ImageJ. Values were averaged (using the four closest neighbors) and plotted as a function of the distance along layer IV parallel to the pia mater. After delineating the borders of immunoreactive patches, their width was measured using ImageJ and compared using Student's *t*-test (GraphPad Prism version 4 for Windows).

## Results

### Expression of MHCI molecules in LGN and V1 of the common marmoset

**Figure 1:**
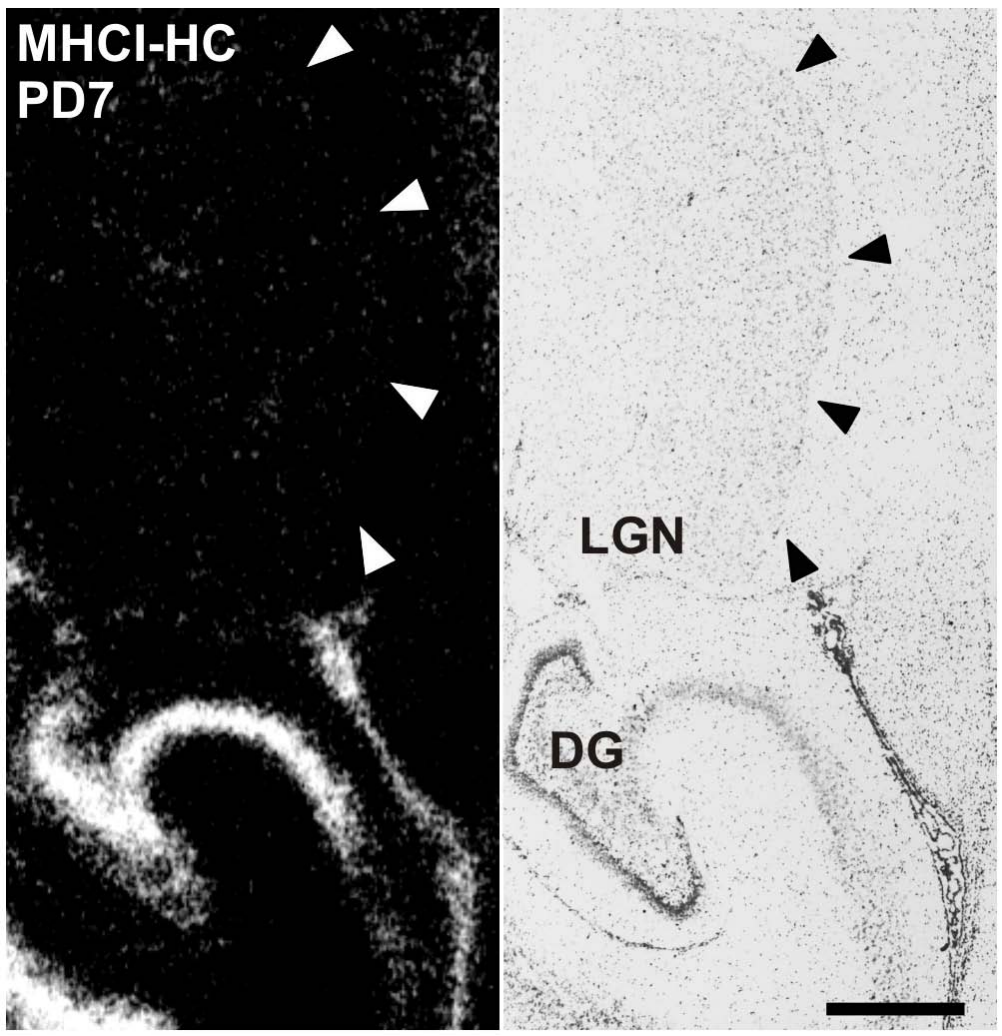
**Lack of MHCI-HC expression in the lateral geniculate nucleus as revealed by *in situ *hybridization**. Autoradiograph of a brain section of a 7 days-old marmoset from the level of the lateral geniculate nucleus (LGN) processed for *in situ *hybridization (left) and toluidine-blue stained section (right). Note the absence of MHCI-HC signals in the LGN (delineated with arrowheads). Abbreviations: Dentate gyrus, DG; postnatal day, PD. Scale bar: 1 mm.

**Figure 2:**
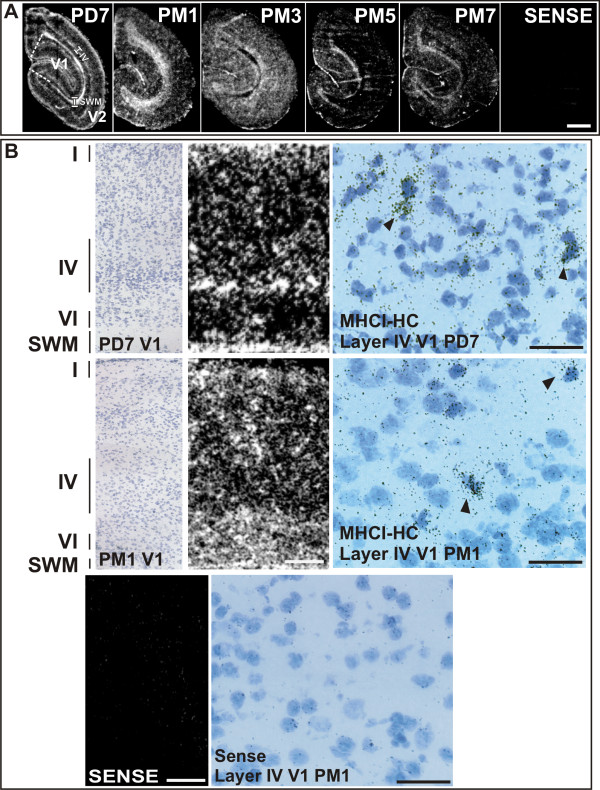
**Expression of MHCI-HC in the primary visual cortex as revealed by *in situ *hybridization**. **A) Autoradiographs of visual cortices processed for *in situ *hybridization**. Sections represent the main stages of visual cortex development. The expression of MHCI-HC in the 7 days-old animal (PD7) decreases with progressing age (compare with 7 months-old animal, PM7). Primary visual cortex (V1) is delineated with white dashed lines. Layer IV and subcortical white matter are delineated with white lines. Abbreviations: Postnatal day, PD; postnatal month, PM; primary visual cortex, V1; secondary/prestriate visual cortex, V2; subcortical white matter, SWM; layer IV, IV. Scale bar: 1 mm. **B) Upper row**: Toluidine-blue stained section of a 7 days-old animal (left) after *in situ *hybridization, and autoradiograph of the same section (middle panel; film autoradiography) reveal MHCI-HC signals in layers I and IV-VI of the primary visual cortex and in the subcortical white matter (SWM). Emulsion autoradiography (right) reveals silver grains clustered over single cells (arrowheads). **Middle row**: Toluidine-blue stained section of a 1-month old animal (left) after *in situ *hybridization and autoradiograph of the same section (middle panel) revealed MHCI-HC signals in all cortical layers and in the subcortical white matter (SWM). Emulsion autoradiography (right) reveals silver grains clustered over single neurons (arrowheads). **Bottom row**: Sense probe revealed only background signals (left; film autoradiograph) and background levels of silver grains in emulsion autoradiography (right). Roman numerals denote cortical layers. Scale bar for film autoradiographs: 1 mm. Scale bar for emulsion autoradiography: 20 μm. Abbreviations: Postnatal day, PD; postnatal month, PM; primary visual cortex, V1; subcortical white matter, SWM.

To investigate the expression of MHCI genes in the marmoset LGN and primary visual cortex, a full-length clone of the heavy chain (HC) of the classical marmoset MHCI gene Caja-G [accession number U59637 [38]) was used for *in situ *hybridization experiments. Animals were chosen based on age and according to the main stages of visual system development [25,26] and were of the following ages: postnatal days 1 and 7; and postnatal months 1, 3, 5, 7, 12, and 21. Although LGN development occurs *in utero *in primates [39,40], we expected to observe strong expression of MHCI-HC (MHCI heavy chain) in newborn animals, as the expression of these genes persists in the LGN of adult rodents and cats [12,15]. To our surprise, there was almost no MHCI-HC signal in the marmoset LGN, even after long exposures to autoradiographic films (Figure 1). However, *in situ *hybridization revealed a strong expression of MHCI-HC throughout the visual cortex. On postnatal day 7, this expression was mainly concentrated in layers I and IV of both primary and secondary visual cortex [V1 and V2, Figure 2A; regions demarcated according to [29,30]) and in the subcortical white matter (Figure 2A and 2B). In older animals (1 to 7 months of age), the signal became more diffuse, with cells in all cortical layers exhibiting MHCI-HC gene expression (Figure 2A and 2B). This pattern of MHCI-HC mRNA expression remained the same throughout postnatal months 12 and 21 (data not shown). Emulsion autoradiography revealed the presence of silver grains clustered over single cells in layer IV of the primary visual cortex (Figure 2B). However, with this technique, the number of labeled cells appeared relatively low because the liquid photo emulsion that is used for dipping the radiolabeled sections is less sensitive to the radioactivity than the autoradiographic films. The sense probe was used as a control and it yielded no signal, thus demonstrating the specificity of the MHCI-HC antisense probe (Figure 2A and 2B).

**Figure 3:**
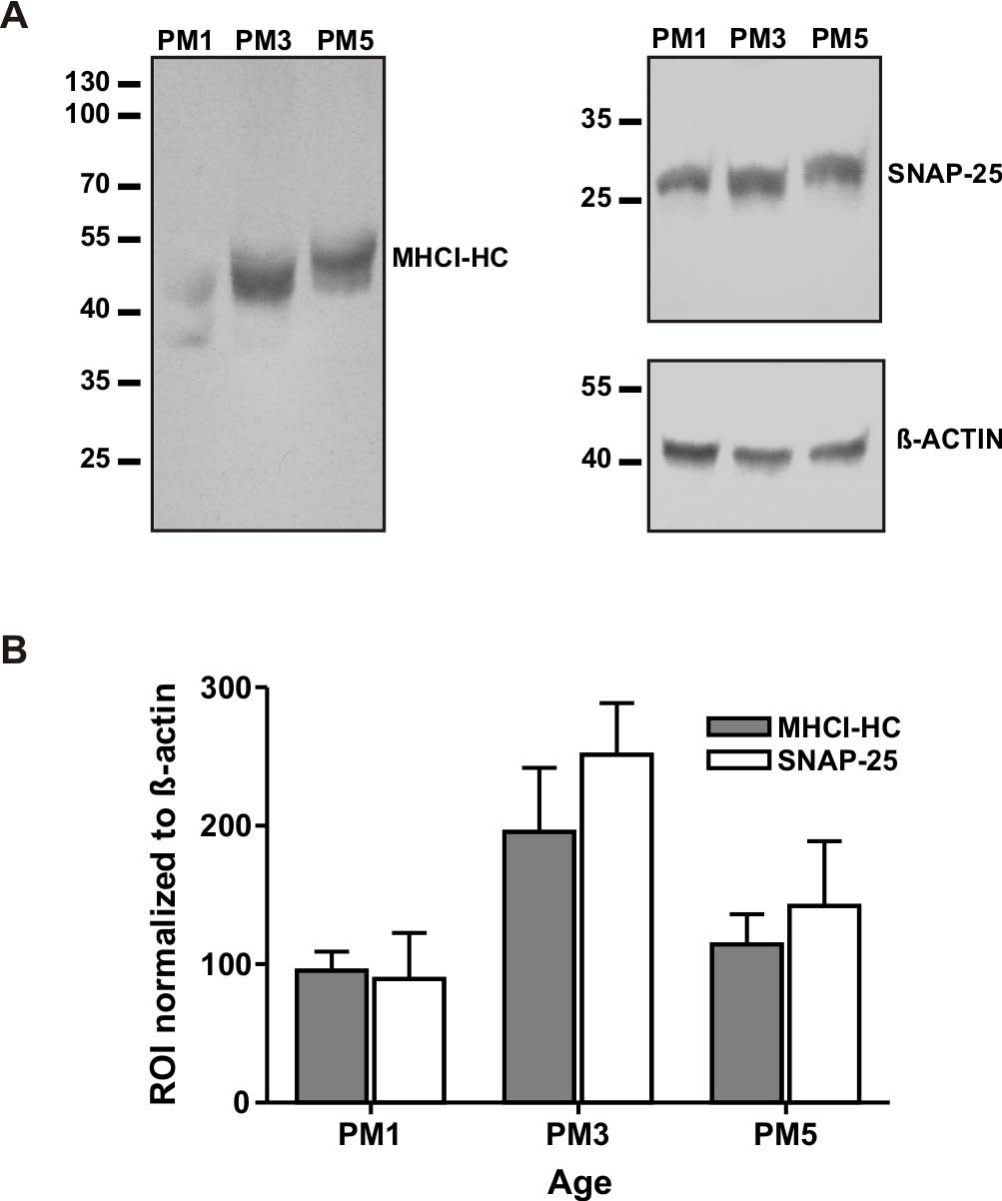
**MHCI-HC protein levels at different stages of visual cortex development**. The antibody TP25.99 (mouse anti-human MHCI) recognized bands of appropriate size for MHCI-HC protein (MHCI-HC, A) in Western blots of proteins extracted from the marmoset visual cortex. Animals were 1, 3 and 5 months old (PM1, 3, 5) representing the main stages of synaptogenesis: initial stage, peak and rapid decline/synaptic refinement, respectively. SNAP-25 was used as a marker of synatogenesis. Data were normalized to ß-actin. Molecular weights (in kDa) are indicated on the left side. Data are from three independent experiments with N = 1 animal per stage (B).

**Figure 4:**
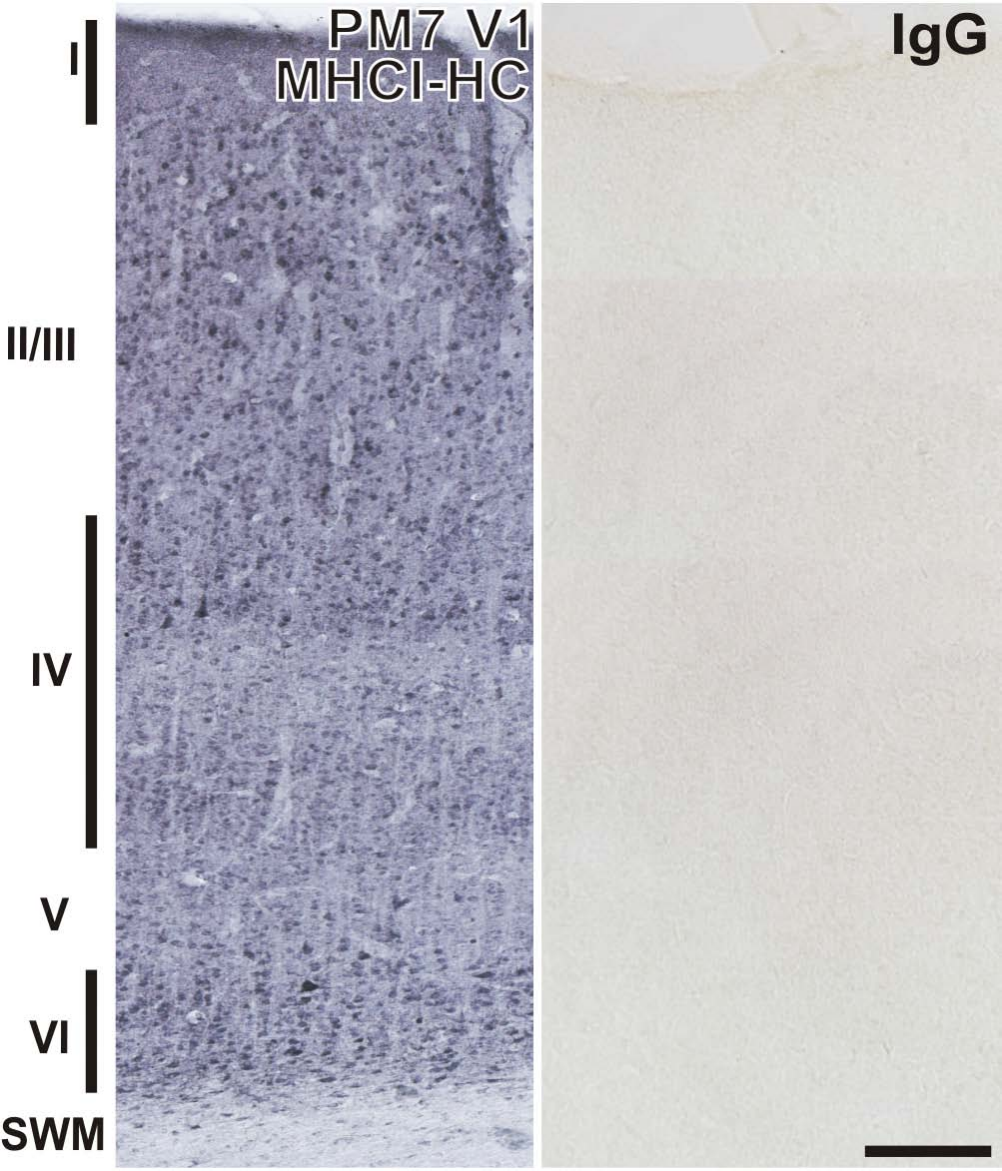
**MHCI-HC immunoreactivity in the primary visual cortex of the common marmoset**. Representative coronal section of the primary visual cortex of a 7 months-old marmoset probed with TP25.99 antibody (mouse anti-human MHCI) revealing strong staining of neurons in all layers (left). Roman numerals denote cortical layers. Control mouse IgG showed no reaction (right). Scale bar: 200 μm. Abbreviations: Postnatal month, PM; subcortical white matter, SWM; primary visual cortex, V1.

Antibodies against marmoset MHCI proteins are not available; however, because of the high similarity of these proteins with their human homologues, we used the well characterized TP25.99 antibody for the detection of marmoset MHCI proteins [34,35]. The epitope to which this antibody binds is situated in the α-3 domain of MHCI molecules, which is monomorphic and the most conserved domain across all species [34]. This domain is almost identical between marmosets and humans. Moreover, the TP25.99 antibody is also one of the antibodies that recognize the free heavy chain form of MHCI [41,42]. TP25.99 recognized a band of approximately 45 kDa on Western blots, which is the expected molecular weight of the MHCI-HC (Figure 3A). Protein expression was quantified in animals aged one, three, and five months, which represent the main stages of synaptogenesis, namely the initial, the peak and the refinement stage [26]. MHCI-HC protein levels at these stages coincided with levels of the synaptogenesis marker SNAP-25 (Figure 3B and [43]). Immunocytochemistry revealed a strong MHCI-HC staining of neurons throughout the primary visual cortex of a 7 months-old animal (Figure 4) and the same staining pattern was observed in all developmental stages (data not shown).

**Figure 5:**
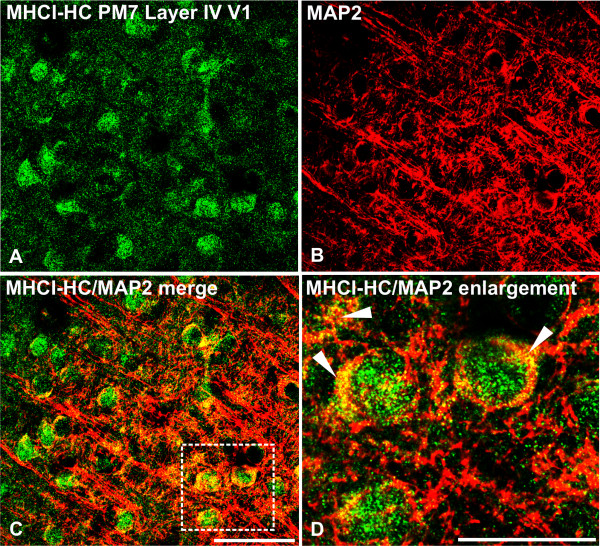
**MHCI-HC protein colocalizes with the neuronal marker MAP-2 in layer IV neurons of the V1**. MHCI-HC (green; A) is localized mainly to the neuronal somata in the primary visual cortex, where it colocalizes with MAP-2 (red; B and C). Higher magnification of the area indicated by the dashed line in C reveals MHCI clustered over MAP2-positive neurons and processes (arrowheads; D). Scale bar in C: 50 μm; scale bar in D: 25 μm. Abbreviations: postnatal month 7, PM7; primary visual cortex, V1.

**Figure 6:**
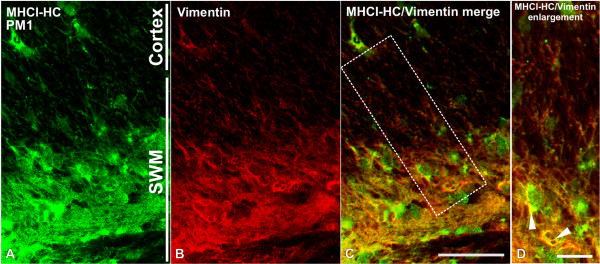
**MHCI-HC colocalizes with radial glia marker in the subcortical white matter of the occipital lobes**. MHCI-HC (green; A) is localized to vimentin-positive radial glia (red; B and C) in the subcortical white matter (SWM). Higher magnification reveals colocalization between MHCI-HC and vimentin signals (white arrowheads in D). Scale bar in C: 50 μm; scale bar in D: 25 μm. Abbreviations: postnatal month 1, PM1; subcortical white matter, SWM.

To further demonstrate that MHCI-HC protein expression in the primary visual cortex is neuronal, we performed double-labeling experiments using TP25.99 and an antibody against microtubule associated protein 2 (MAP2), which is an established dendritic marker [44-46]. A punctuate pattern of MHCI-HC immunoreactivity that colocalized with MAP2-positive neuronal processes was observed in the neuronal somata, dendrites and neuropil in the main thalamorecipient layer of V1, layer IV (Figure 5). As previously reported [23], MHCI-HC protein partially colocalized with both gephyrin and SAP102, markers of inhibitory and excitatory synapses respectively ([47-50]; Additional file 3). *In situ *hybridization also showed strong MHCI-HC gene expression in the subcortical white matter in the occipital lobes, consistent with previous studies on MHCI in the visual cortex of cats [15]. This region contains specialized glial cells, radial glia, involved in neuronal differentiation and migration [51]. Double-labeling experiments using antibodies against MHCI-HC and vimentin, a marker of radial glia [52,53] confirmed that MHCI-HC is indeed expressed on radial glia in the subcortical white matter (Figure 6). Vimentin-positive cells also displayed strong immunoreactivity for GFAP (glial fibrillary acidic protein) in the subcortical white matter ([54]; Additional file 4), but vimentin immunoreactivity could only be detected in this region. No vimentin-positive cells and only GFAP immunoreactivity was detected in the visual cortex (Additional file 4). The MHCI-HC signal in the V1 did not colocalize with GFAP-positive astrocytes (Additional file 4). However, fully assembled heterotrimeric MHCI molecules could be detected on microglial cells in the cortex with W6/32 antibody (Additional file 5). W6/32 is a prototypic anti-MHC class I antibody that binds to a conformational epitope on MHCI heavy chains upon their association with β-2-microglubulin and the peptide [36,41,55]. Interestingly, another antibody that recognizes free heavy chains, monoclonal Q1/28 [34] recognized neuronal-like processes and cell bodies in the marmoset cortex (Additional file 5). This, along with our previous studies [56,57], further suggested that neurons might express MHC class I molecules preferentially in their free heavy chain form on the cell surface. Unfortunately, we could not reliably detect ß-2-microglobulin subunit with the antibodies available to us (data not shown), although we have detected ß-2-microglobulin transcripts in the marmoset cortex in our previous study [56].

### Expression levels of MHCI molecules are regulated by neuronal activity

**Figure 7:**
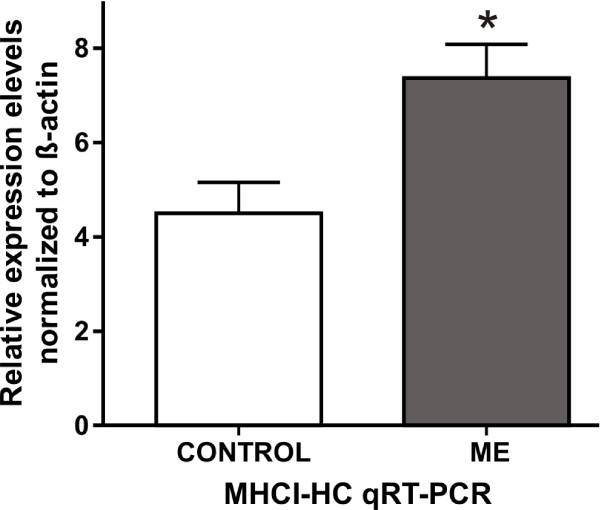
**MHCI-HC mRNA expression is upregulated in response to monocular enucleation**. qRT-PCR reveals a significant difference in MHCI-HC mRNA expression levels in the whole visual cortices of animals that have undergone monocular enucleation (ME) and controls. Data are expressed as mean ± SEM (standard error of the mean) and are representative of three independent experiments performed with samples isolated from both hemispheres of N = 3 animals/group. Significant differences between groups as determined by Student's two-tailed *t*-test: *, p < 0.05 (t = 2.961 df = 10).

**Figure 8:**
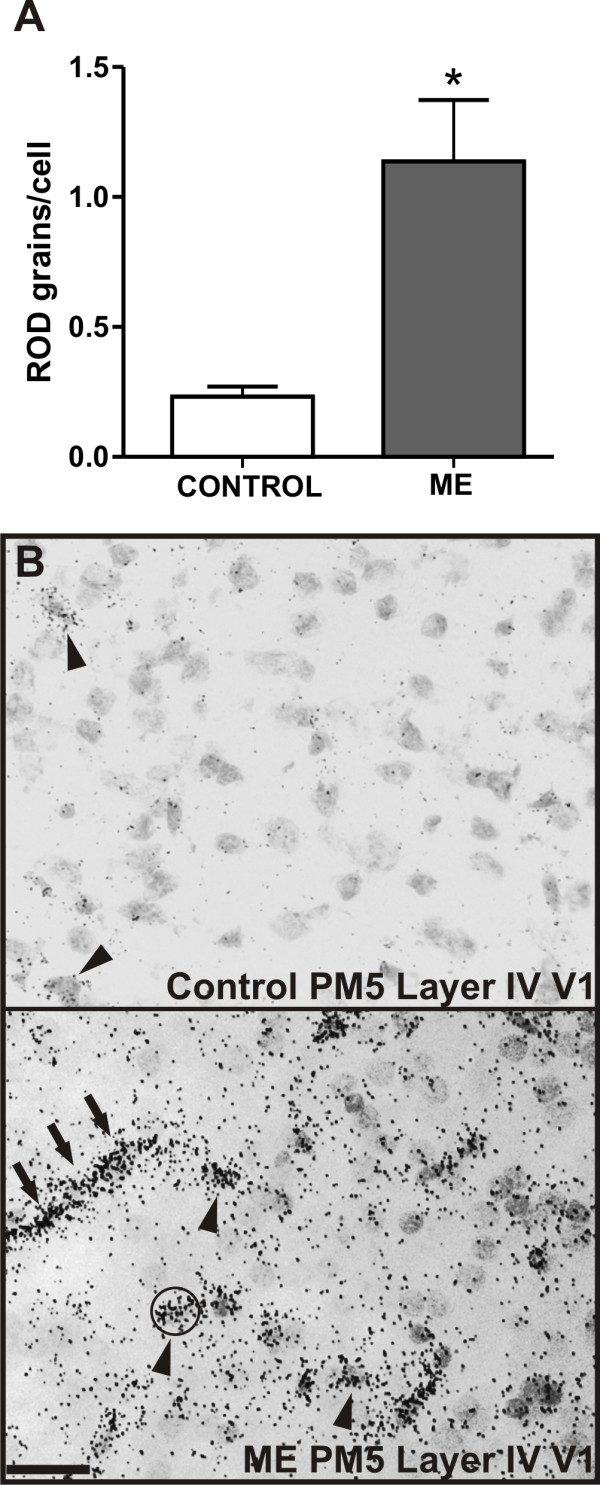
**Monocular enucleation upregulates MHCI-HC mRNA in layer IV of the primary visual cortex**. **A: **Numbers of silver grains per cell reveal elevated levels of MHCI-HC expression in layer IV of the V1 of enucleated animals (ME). Data are expressed as mean ± SEM (standard error of the mean), N = 3 animals/group. Significant differences between groups as determined by Student's two-tailed t-test: *, p < 0.05 (t = 3.781, df = 4). **B: **Examples of sections from layer IV of the primary visual cortex of a control and an enucleated animal showing silver grains over cells (circular counting mask). Scale bar: 20 μm. Abbreviations: Postnatal month 5, PM5; primary visual cortex, V1.

Several previous studies suggested a variety of nonimmune roles for MHCI molecules, most of which refer to involvement of MHCI free heavy chain form in receptor trafficking, cell growth and differentiation [7]. As the temporal and spatial pattern of MHCI expression correlates with synaptogenesis in the primary visual cortex, we decided to study this further. Enucleation of one eye before critical stages of visual cortex development induces significant morphological and physiological changes in V1 [18,22]. Monocular deprivation, induced by either unilateral enucleation or by other means (eye-lid suture or pharmacological blockade of retinal activity), causes the intact eye to dominate the V1, both anatomically and physiologically [18]. Dendritic arbors of the neurons in V1 receiving afferents from the deprived eye shrink [58]. This shrinkage presumably reduces the width of ODCs subserving the deprived eye [18,58,59]. On the other hand, ODCs subserving the intact eye expand [60-63]. It is known that synaptic input regulates gene expression in target neurons and previous gene expression studies have reported major activity-dependent changes in the expression of numerous genes [64,65]. We used monocular enucleation (ME) to induce the aforementioned changes in V1 of the marmoset. Animals were enucleated at one month and were sacrificed at five months of age [28]. MHCI-HC gene expression in visual cortices of monocularly enucleated animals and age-matched controls were compared using qRT-PCR with MHCI-HC PCR primers designed to recognize the intracellular domain of Caja-G. Both left and right visual cortices were used from all animals. In the present study, MHCI-HC transcript levels were significantly higher in the visual cortices of enucleated animals, as determined by qRT-PCR (quantitative Real Time Polymerase Chain Reaction, Figure 7). Also, quantitative *in situ *hybridization using the Caja-G probe confirmed the qRT-PCR results revealing a higher density of silver grains over cells in layer IV of the primary visual cortex of the enucleated animals (Figure 8A and 8B).

### Neurons with higher MHCI-HC expression receive afferents from the intact eye

**Figure 9:**
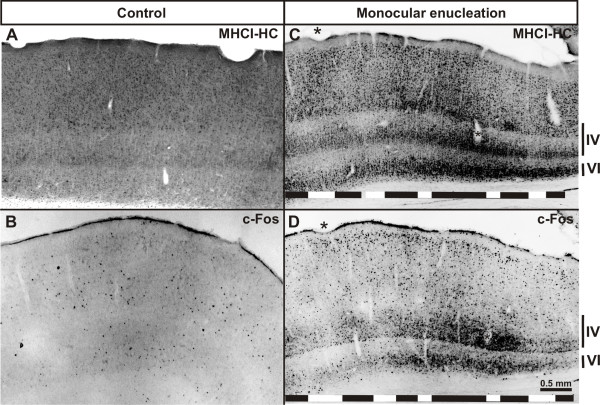
**Primary visual cortex reveals a banded pattern of MHCI-HC and c-Fos immunoreactivity in enucleated animals**. **A-D: **Patches of stained neurons can be observed in enucleated (C and D), but not in control animals (A and B). These patches are detected with the MHCI antibody (C) and with the c-Fos antibody (D). Asterisks in C and D denote identical blood vessels. Roman numerals indicate cortical layers. Abbreviation: primary visual cortex, V1; monocularly enucleated animal, ME. **Note: **Black horizontal lines in the lower parts of C and D indicate areas with high MHCI-HC and c-Fos immunoreactivity; white lines point to low immunoreactivity areas.

**Figure 10:**
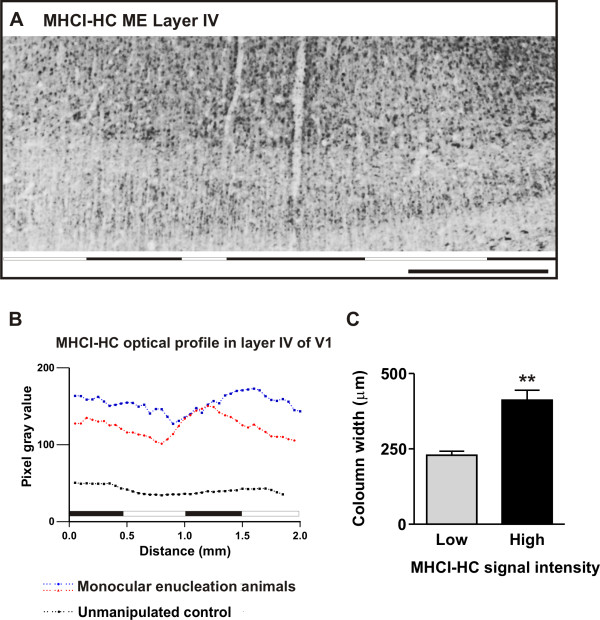
**MHCI-HC is upregulated in primary visual cortex neurons that receive afferents from the intact eye**. **A: **Example of the region analyzed for optical density measurements (delineated with white, dashed line). Abbreviations: primary visual cortex, V1; monocularly enucleated animal, ME. **B: **Optical density profiles recorded from layer IV of the primary visual cortex of a control animal (black dotted line) and two enucleated animals (red and blue dotted lines). **C: **Width of MHCI-HC immunoreactive column-like patches showing that the wider columns display stronger MHCI-HC staining indicating that MHCI-HC levels are elevated in neurons receiving afferents from the intact eye. Difference between the groups is highly significant as determined by Student's two tailed *t*-test: **, p < 0.01 (t = 5.166 df = 4). Data are expressed as mean ± SEM (standard error of the mean), N = 3 animals/group, 12 measurements per animal. **Note: **Black horizontal lines in (A) and (B) indicate areas with high MHCI-HC immunoreactivity, while white lines point to low immunoreactivity areas.

*In situ *hybridization was not able to shed light as to where the elevated MHCI-HC transcript levels are localized, in neurons receiving afferents from the intact or from the enucleated eye. Hence, in order to localize MHCI-HC expression in the V1 of the enucleated animals, we performed immunohistochemistry using the TP25.99 antibody. Control animals (5 months old) displayed a relatively uniform staining pattern throughout V1 (Figure 9A). In contrast, TP25.99 revealed a patchy pattern of immunoreactivity in V1 of enucleated animals (Figure 9Band Additional file 6). To assign the regions of high and low immunoreactivity to neurons receiving afferents from either the intact or the enucleated eye, we stained adjacent sections for a known activity-marker, a method commonly employed in monocular deprivation paradigms [66,67]. For this study, we used c-Fos as a marker of neuronal activity [68,69]. Expression of both c-Fos and MHCI-HC was uniform, albeit at a very low level in control animals (Figure 9A and 9B). However, in animals that had undergone monocular enucleation, column-like patches of c-Fos and MHCI-HC immunoreactivity were visible throughout layer IV of V1 (Figure 9C and 9D and Additional file 6). Comparison of adjacent sections stained for MHCI-HC and c-Fos showed that the columns with high MHCI-HC immunoreactivity overlap with regions of high c-Fos immunoreactivity (Figure 9C and 9D and Additional file 6). Secondary visual cortex (V2) displayed no such column-like patches, neither in c-Fos nor in MHCI-HC staining (data not shown). Although the patchy appearance of MHCI-HC immunoreactivity was encompassing not only layer IV, but also layers II and III, we measured the optical density profiles only in layer IV since this is the main recipient layer. In sections from the enucleated animals, the MHCI-HC optical density profile from the entire layer IV in V1, measured along its horizontal axis (Figure 10A), displayed variations in intensity that were not observed in control animals (Figure 10B). Regions with high and low immunoreactivity were detected in these subjects. In contrast, control animals displayed a relatively weak and uniform staining of all layers (Figure 9A; Figure 10B). As it was previously shown that columns with neurons receiving afferents from the intact eye are wider and occupy larger regions in V1, we measured the width of columns exhibiting high and low MHCI-HC immunoreactivity [61]. A significant difference was found between the widths of columns exhibiting high and low immunoreactivity, with stronger MHCI-HC staining intensity localized in the wider columns (Figure 10C). Furthermore, a number of previous studies have associated the expression of NMDAR1 (*N*-methyl *D*-aspartate receptor subunit 1) with developmental plasticity in the visual cortex [37,61,70]. After monocular deprivation, higher levels of NMDAR1 are present on neurons that still receive visual input and afferents from the intact eye [37,61,70]. In the present study, MHCI-HC protein was localized on NMDAR1-positive neurons in V1 of enucleated animals, which further supports the notion that MHCI-HC expression levels are associated with neuronal activity (Additional file 7). Interestingly, transcript levels of the activity marker c-Fos [68,69] were also significantly elevated in the enucleated animals, similar to MHCI-HC levels (Additional file 8). These results confirm the hypothesis that the columns with high MHCI-HC immunoreactivity receive afferents from the intact eye, suggesting that high expression levels of this gene are associated with high neuronal activity.

## Discussion

Several recent studies have implicated MHCI proteins in the elimination of excess synapses in the visual system during distinct phases of cortical development [12,15,23]. MHCI molecules, as well as the organization of the visual system, differ between rodents and primates, including humans [17,24]. We therefore investigated the expression of neuronal MHCI molecules in the brain of the common marmoset monkey. To the best of our knowledge, the present study is the first to characterize the spatio-temporal pattern of MHCI gene expression in the primary visual cortex during postnatal development in a nonhuman primate.

### Expression and localization of MHCI molecules in V1

It is known that virtually all nucleated cells express MHCI molecules in form of completely assembled heterotrimers, and in some cases in form of the peptide-free heavy chains [4,6]. The present study clearly showed that MHCI molecules are expressed on MAP-2 positive neurons of V1. However, in this and our previous studies [56,57], we were only able to detect neuronal expression of MHCI heavy chain proteins. In addition, *in situ *hybridization and immunohistochemistry revealed high expression levels of MHCI-HC throughout the subcortical white matter (SWM) of the occipital lobes, consistent with what has previously been observed in cats [15]. In SWM, MHCI-HC protein colocalized with vimentin-positive radial glial cells. These highly specialized cells are regarded as neuronal precursors and are involved in proper development and migration of cortical neurons [51]. In primates, these cells are also GFAP-positive from early prenatal stages, but can be distinguished from astrocytes on the basis of their distinct morphology and localization [71]. We did not detect any vimentin-positive cells in the cortical layers, and MHCI-HC was not expressed in GFAP-positive cortical astrocytes (Additional file 4), although these astrocytes were stained with the antibody that recognized fully assembled, heterotrimeric form of MHCI (Additional file 5). These findings further implicate MHCI in proper development of the visual cortex, although their potential function in this context remains elusive [72]. As the marmoset MHCI gene cluster is not fully characterized yet, we hope that future studies will shed light on the exact identity and potential functional differences between neuronal, radial glial and microglial MHCI.

Previous studies have shown that MHCI proteins may be localized to both pre- and postsynaptic sites [14,23,57,73,74]. The MHCI-HC molecules detected here with confocal microscopy and TP25.99 antibody seem to be associated with the postsynaptic sites (cell bodies and dendrites, Figure 5) and they displayed no obvious preference for excitatory *vs *inhibitory synapses (Additional file 3), as has previously been reported [23].

### MHCI-HC mRNA and protein expression in the developing visual cortex

In contrast to the previous studies in rodents and cats [12,15], we did not detect expression of MHCI-HC in the LGN at postnatal stages used in this study. It is possible that MHCI genes are expressed prenatally in the marmoset LGN, as the segregation of LGN layers in primates occurs *in utero *[39,40]. Furthermore, the probe used here for the detection of MHCI-HC transcripts is specific for a subset of MHCI molecules, while the probes used in previous studies were mostly pan-specific and therefore detected a larger variety of MHCI molecules [15]. Even so, the present data showed that MHCI genes are strongly expressed throughout all regions of the visual cortex of the marmoset. In contrast to the *in situ *hybridization that revealed a distinct spatio-temporal pattern of MHCI-HC expression, the staining pattern generated with the TP25.99 antibody was rather uniform throughout all cortical layers and developmental stages. While the MHCI subset-specific riboprobe used for *in situ *hybridization very likely targeted only a few MHCI genes, the antibody we used for histochemical detection of MHCI targets the most conserved, monomorphic part of these molecules. Hence, the TP25.99 antibody likely recognized a large number of MHCI-HC proteins expressed on V1 neurons, including the ones targeted at the mRNA level by *in situ *hybridization riboprobe. As previously mentioned, the common marmoset MHCI gene cluster is not yet fully characterized and so far only two loci have been described, the classical Caja-G and the non-classical Caja-E [38]. Based on this (Additional file 2) and our previous studies [56,57], we believe that the neuronal MHCI-HC detected in the present study is a classical marmoset MHCI, Caja-G. Nonetheless, the levels of MHCI-HC protein expression in the visual cortex correlated well with the levels of SNAP-25 (synaptosome-associated protein 25 kDa). This protein is an established synaptogenesis marker [43,75,76] and the present data on SNAP-25 protein expression levels confirmed previous findings that synapse density in the marmoset V1 reaches very high values in the third postnatal month, followed by a rapid decline due to synapse elminiation [25,26]. Contrary to our expectations and to the proposed role for MHCI in the elimination of synapses [12,15,23], MHCI-HC protein expression was at its peak already in the third postnatal month, together with SNAP-25 levels, and was in decline in the fifth postnatal month. This suggests that, as recently proposed [74], MHCI proteins may exert their function during synaptogenesis, rather than during refinement and elimination of synapses.

### MHCI expression in the visual cortex is activity dependent

As genetic manipulation of primates is still either unavailable or at an early stage [77], we used monocular deprivation as an alternative model to further clarify the role of MHCI molecules in the marmoset visual cortex. Prolonged monocular deprivation induced by various means (in the order of months) induces morphological changes in the visual cortex because neurons that receive input from the intact eye expand their connections and cortical space at the expense of neurons that receive afferents from the deprived eye [21,60]. A number of studies have shown that monocular deprivation results in the upregulation of growth factors and genes associated with neuronal degeneration or synaptogenesis [64,65,78]. In mice, monocular deprivation increases levels of SNAP-25 and SynCAM (synaptic cell adhesion molecule) transcripts [78], both of which are required for synaptogenesis [79-81]. Interestingly, MHCI-HC transcript levels detected in the present study after monocular enucleation with qRT-PCR and *in situ *hybridization for classical marmoset MHCI-HC were also higher in response to monocular enucleation. The levels of neuronal activity marker c-Fos were also elevated. On the contrary, GFAP displayed no significant change in either expression levels or in expression pattern in enucleated animals (Additional file 9). This even further correlated MHCI-HC levels with neuronal activity, and not with microglial activation.

We used immunohistochemistry to localize the elevated MHCI-HC levels to neurons receiving afferent inputs from the intact eye. In V1 of enucleated animals, both the well-established activity marker c-Fos [68,69] and MHCI-HC immunostaining revealed a pattern of column-like patches, where regions of high c-Fos and high MHCI-HC immunoreactivity overlapped and were wider than regions with low immunoreactivity. These changes were visible not only in layer IV, but also in layers I-III and layer VI, which is not surprising since it is known that prolonged deprivation causes responses in neurons in all cortical layers [82]. Previous studies in marmosets have shown that such patches correspond to ocular dominance columns. They differ in size in monocularly deprived animals, with wider ODCs receiving input from the intact eye and showing higher levels of NMDAR1 [61,70]. Interestingly, MHCI-HC protein detected in this study also localized to NMDAR1-positive neurons in the cortex of enucleated animals (Additional file 7), which is also indicative of MHCI association with neuronal activity [61,70]. Therefore, MHCI-HC immunoreactivity in the visual cortex of enucleated animals suggested the upregulation of expression of this class of genes in neurons receiving input from the intact eye. As mentioned above, synaptogenesis in the marmoset visual cortex peaks in the third postnatal month. Although elimination of synapses and synaptogenesis may occur within the same time window in the developing V1 [83], it is interesting to note that MHCI expression peaks during the synaptogenesis stage. Moreover, the observed upregulation of MHCI levels in response to monocular enucleation points to a possible role in synaptogenesis.

### Potential interspecies differences in properties of neuronal MHCI molecules

The so-called classical MHCI molecules detected in this study are traditionally regarded as having only immunity-related functions, but they have recently been implicated in synaptic refinement in the visual system [23]. Even though our data indicated a potential interspecies difference in the function of classical MHCI in the visual system, the very large number of these genes and the fact that there are no true orthologues between rodents and primates [17] makes direct comparisons very difficult. On the other hand, the possible interspecies differences in function are not surprising. MHCI molecules exhibit a multitude of functions and may, for example, transmit both activating and inhibitory signals in the immune system [7,84]. MHCI proteins are also necessary for immunological synapse formation [85]. In addition to their role in synaptic refinement and stripping, MHCI molecules have been implicated in stabilization of certain types of terminals in the peripheral nervous system of mice [86]. The exact mechanism and role of MHCI in the visual cortex is yet to be elucidated, although previous studies have shown that MHCI may associate with PI-3, which is known to be a synaptogenic molecule [8,87]. Another possibility includes the MHCI-presenting peptides, as it is known that they reflect the metabolic state of the cell [88]. Given that neuronal MHCI expression in the visual cortex is activity-regulated, similar to c-Fos ([15] and this study), this would enable it to mark or tag neurons or neuronal populations that display a distinct activity. A recent study also suggested that neuronal MHCI may be involved in NMDA-induced internalization of AMPARs (α-amino-3-hydroxyl-5-methyl-4-isoxazole-propionate receptors, [89]). AMPARs belong to the group of activity-regulated molecules involved in developmental plasticity in the visual cortex [90,91]. As we were able to reliably detect only the free heavy chain form of MHCI proteins on neurons (this study and [56,57]), the suggested link between MHCI and trafficking of AMPARs is very intriguing considering our current study and previous studies on MHCI involvement in receptor trafficking [6,56,57].

## Current limitations

As previously mentioned, there are no true orthologues between MHC class I genes of rodents and primates [17]. MHC gene cluster shows significant interspecies variability, as well as a fast evolutionary rate [2,3,17]. This makes drawing parallels between rodent and primate studies extremely difficult. Furthermore, current policies on the use of non-human primates in research are very limiting, which makes functional studies on non-human primates hard to perform.

It is our hope that the further development of transgenic marmoset models [77], more flexible research policies, as well as detailed characterization of the marmoset MHC cluster, will provide future researchers with the tools to investigate the potential functional differences between neuronal MHCI molecules in rodents and primates in more detail.

## Conclusions

Despite the possible interspecies differences in expression of MHCI molecules in the brain, all studies on this topic have highlighted yet another important role of these molecules. MHC genes have been linked to a number of CNS disorders and the discovery of their roles in plasticity processes in the brain has shed new light on their etiology [84,92-94]. Further research is warranted to elucidate the exact mechanisms of action of the neuronal MHCI genes and the exact identity of the neuronal MHCI gene possibly involved in synaptogenesis.

## Competing interests

The authors declare that they have no competing interests.

## Authors' contributions

AR designed and carried out the study, conducted statistical analyses, and drafted the manuscript. CS and KMR performed animal surgeries. GF, LW and EF participated in the overall study design and helped draft the manuscript. All authors read and approved the final manuscript.

## Supplementary Material

Additional file 1 **All products of the qRT-PCR reactions yielded a single product**. After the qRT-PCR reactions, 3-5 μL of one sample for a primer pair (Caja-G, c-Fos, GFAP and ACTB) was run on a 2.5% agarose gel. No template controls (NTC) revealed no product. DNA size marker (NEB QuickLoad 50 bp) with relevant sizes in bp marked on the left side of the gel images. Click here for file

Additional file 2 **Caja-G qRT-PCR yielded a single product corresponding to Caja-G locus only**. After the qRT-PCR, the reaction product was purified using QIAquick PCR Purification Kit (QIAGEN) and sequenced using the forward primer used for the qRT-PCR reaction (bold and italics in the Caja-G sequence). The obtained sequence (bolded and highlighted in grey in the Caja-G sequence) was identified as Caja-G (alleles Caja-G*1, *3 and *5) using NCBI BLAST tool. Example of pairwise alignments generated by BLAST, as well as the BLAST hits table are shown in the lower part of the figure. Click here for file

Additional file 3 **MHCI-HC protein is localized on both excitatory and inhibitory synapses in the marmoset primary visual cortex**. **Upper row: **MHCI-HC (green; A) partially colocalizes with inhibitory synapse marker gephyrin (red, B; white arrowheads, C). **Lower row: **MHCI-HC (green; D) partially colocalizes with the excitatory synapse marker SAP102 (red, D; white arrowheads, E). Scale bar for all images: 20 μm. Click here for file

Additional file 4 **MHCI-HC protein is not detected on glial cells in the cortex**. **Upper panel: **vimentin-positive cells and processes (green) in subcortical white matter of the occipital lobes are also GFAP-positive (red; white arrowheads in merged image). Scale bar: 50 μm. **Lower panel: **MHCI-HC positive cells (green) in subcortical white matter of the occipital lobes are also GFAP-positive (red; white arrowheads in merged image). Abbreviations: SWM, subcortical white matter; PM1, postnatal month 1. Scale bar: 50 μm. **A) Upper panel: **vimentin immunoreactivity cannot be detected in the cortex, as opposed to GFAP-immunoreactivity (red). Scale bar: 30 μm. **Lower panel: **In the layer IV of the visual cortex, MHCI-HC signal (green) is not overlapping with GFAP-positive astrocytes (red; merged image). Scale bar: 30 μm. Click here for file

Additional file 5 **Microglial MHCI molecules are heterotrimeric**. **Left panel: **Immunohistochemistry with W6/32 antibody specific for the heterotrimeric form of MHCI molecules revealed staining of microglial cells (arrows) and processes in the marmoset cortex. **Middle panel: **Q1/28 antibody, which recognizes the free heavy chain form of MHCI molecules, labelled neurons in the same region (arrowheads), similar to what can be detected with TP25.99 antibody (right panel, arrowheads). Scale bar for all images: 150 μm. Click here for file

Additional file 6 **Primary visual cortex of all enucleated animals reveals banded pattern of MHCI-HC and c-Fos immunoreactivity**. Black horizontal lines in the lower parts of the images indicate areas with high MHCI-HC and c-Fos immunoreactivity; white lines point to low immunoreactivity areas. Animals ME1 and 2 are siblings. Asterisks denote identical blood vessels. **Note: **Middle panel, MHCI-HC staining: black region in the right part of the image is an artefact. **Note: **Black horizontal lines in upper panel in (B) indicate areas with high c-Fos immunoreactivity, while white lines point to low immunoreactivity areas. Scale bar: 0.5 mm. Click here for file

Additional file 7 **MHCI-HC protein is localized on NMDAR1-positive neurons in the visual cortex of enucleated animals**. MHCI-HC (A) is present on NMDAR1-positive neurons in the primary visual cortex of enucleated animals (B, white arrowheads in C). Higher magnification reveals MHCI-HC clusters (D) overlapping with NMDAR1 clusters (E, white arrowheads in F). Scale bar in C: 50 μm; scale bar in D: 25 μm. Abbreviations: ME, monocularly enucleated animals; V1, primary visual cortex. Click here for file

Additional file 8 **c-Fos mRNA expression is upregulated in response to monocular enucleation**. qRT-PCR reveals a significant difference in c-Fos mRNA expression levels in the whole visual cortices of animals that have undergone monocular enucleation (ME) and controls. Data are expressed as mean ± SEM (standard error of the mean) and are representative of three independent experiments performed with samples isolated from both hemispheres of N = 3 animals/group. Significant differences between groups as determined by Student's two-tailed *t*-test: *, p < 0.05 (t = 2.875 df = 10). Click here for file

Additional file 9 **Effects of monocular enucleation on the expression of GFAP mRNA and protein expression**. **A) Enucleation has no significant effect on the levels of GFAP mRNA in the visual cortex as determined with qRT-PCR**. Abbreviations: ME, monocularly enucleated animals. Data are expressed as mean ± SEM (standard error of the mean) from 2 independent experiments, N = 3 animals/group (ns, not significant; Student's two-tailed t-test). **B) Enucleation has no visible effect of GFAP immunoreactivity in the visual cortex of enucleated animals**. Adjacent sections were processed for c-Fos and GFAP immunostaining. While c-Fos revealed patchy immunoreactivity in the visual cortex of enucleated animals (upper panel), GFAP revealed uniform staining pattern (lower panel). Abbreviations: ME, monocularly enucleated animals; V1, primary visual cortex. Click here for file
