# Supplementary figures and images for: Activity-dependent regulation of MHC class I expression in the developing primary visual cortex of the common marmoset monkey
Source: Behav Brain Funct. 2011 Jan 4;7:1. doi: 10.1186/1744-9081-7-1 (PMC3023691; doi:10.1186/1744-9081-7-1)

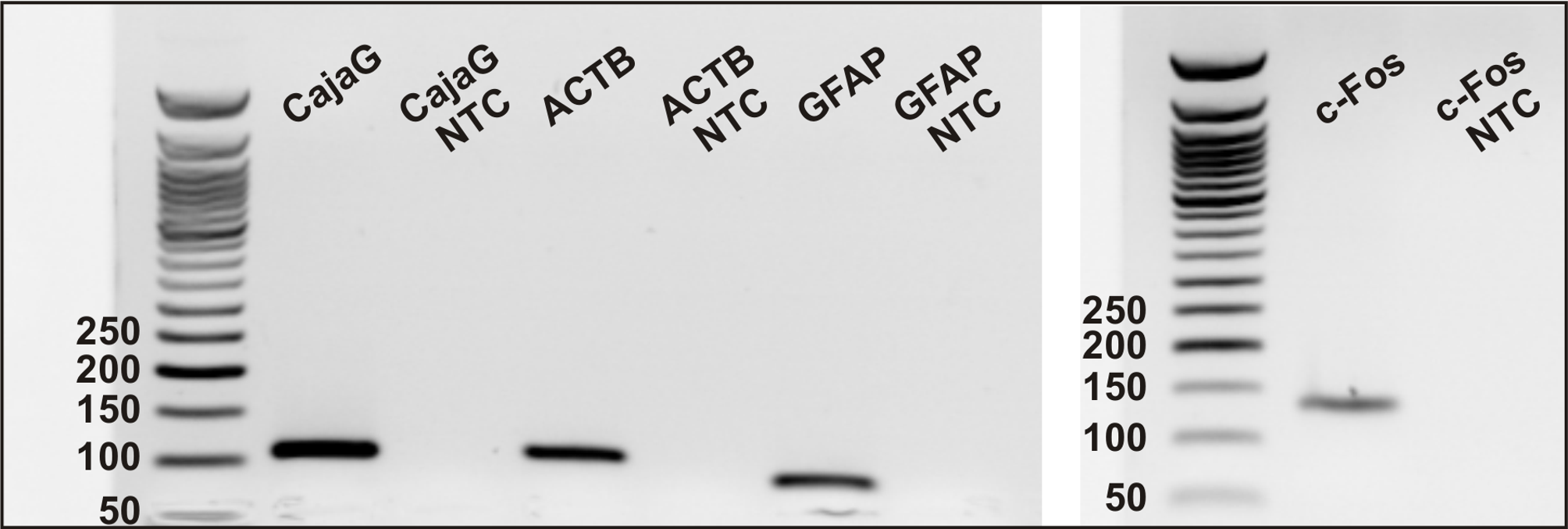

Supplement: Additional file 1 — All products of the qRT-PCR reactions yielded a single product. After the qRT-PCR reactions, 3-5 μL of one sample for a primer pair (Caja-G, c-Fos, GFAP and ACTB) was run on a 2.5% agarose gel. No template controls (NTC) revealed no product. DNA size marker (NEB QuickLoad 50 bp) with relevant sizes in bp marked on the left side of the gel images. [file 1744-9081-7-1-S1.PNG]

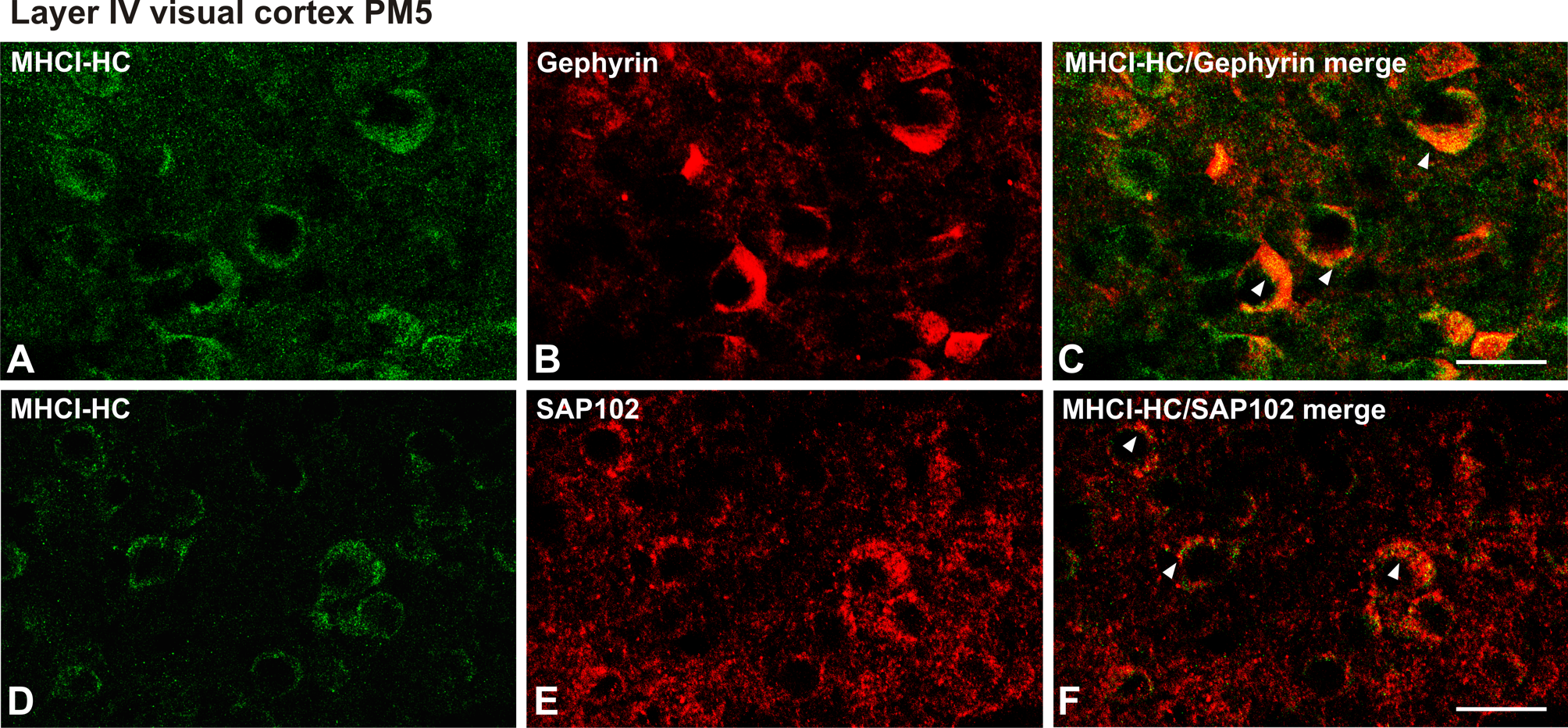

Supplement: Additional file 3 — MHCI-HC protein is localized on both excitatory and inhibitory synapses in the marmoset primary visual cortex. Upper row: MHCI-HC (green; A) partially colocalizes with inhibitory synapse marker gephyrin (red, B; white arrowheads, C). Lower row: MHCI-HC (green; D) partially colocalizes with the excitatory synapse marker SAP102 (red, D; white arrowheads, E). Scale bar for all images: 20 μm. [file 1744-9081-7-1-S3.PNG]

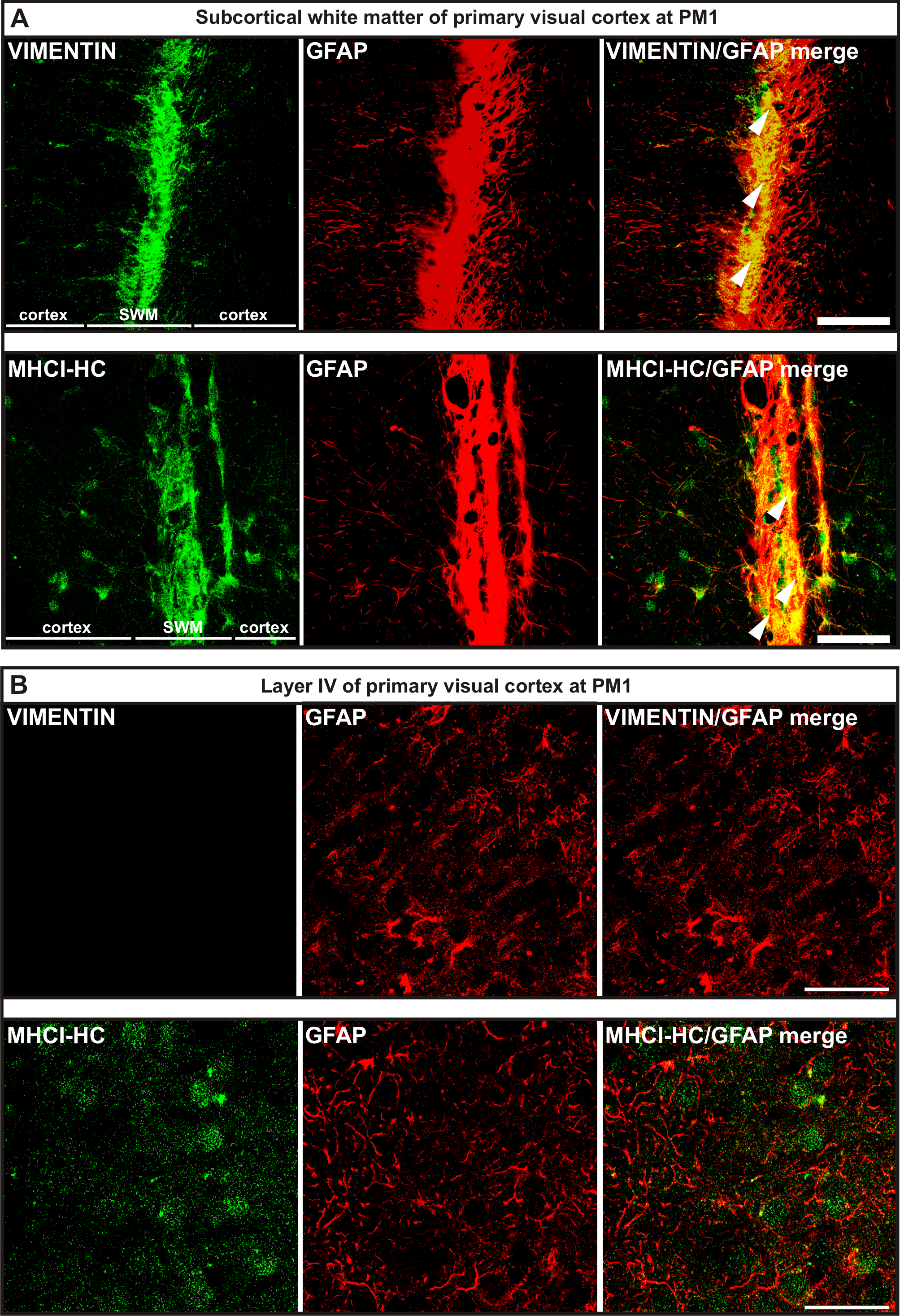

Supplement: Additional file 4 — MHCI-HC protein is not detected on glial cells in the cortex. Upper panel: vimentin-positive cells and processes (green) in subcortical white matter of the occipital lobes are also GFAP-positive (red; white arrowheads in merged image). Scale bar: 50 μm. Lower panel: MHCI-HC positive cells (green) in subcortical white matter of the occipital lobes are also GFAP-positive (red; white arrowheads in merged image). Abbreviations: SWM, subcortical white matter; PM1, postnatal month 1. Scale bar: 50 μm. A) Upper panel: vimentin immunoreactivity cannot be detected in the cortex, as opposed to GFAP-immunoreactivity (red). Scale bar: 30 μm. Lower panel: In the layer IV of the visual cortex, MHCI-HC signal (green) is not overlapping with GFAP-positive astrocytes (red; merged image). Scale bar: 30 μm. [file 1744-9081-7-1-S4.PNG]

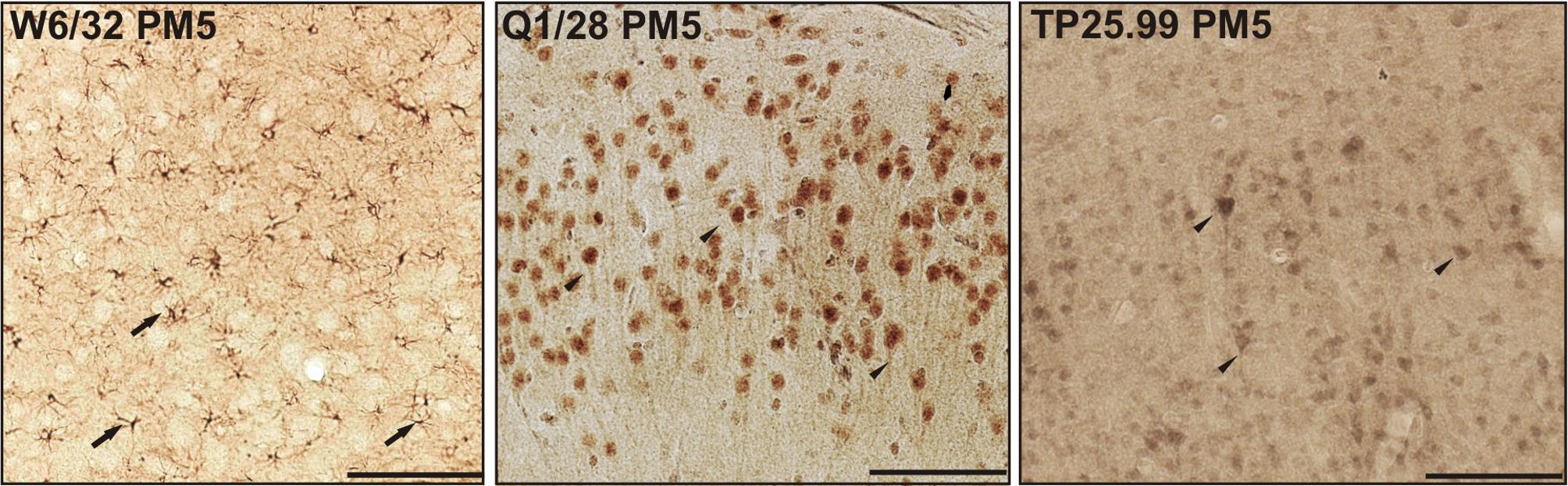

Supplement: Additional file 5 — Microglial MHCI molecules are heterotrimeric. Left panel: Immunohistochemistry with W6/32 antibody specific for the heterotrimeric form of MHCI molecules revealed staining of microglial cells (arrows) and processes in the marmoset cortex. Middle panel: Q1/28 antibody, which recognizes the free heavy chain form of MHCI molecules, labelled neurons in the same region (arrowheads), similar to what can be detected with TP25.99 antibody (right panel, arrowheads). Scale bar for all images: 150 μm. [file 1744-9081-7-1-S5.PNG]

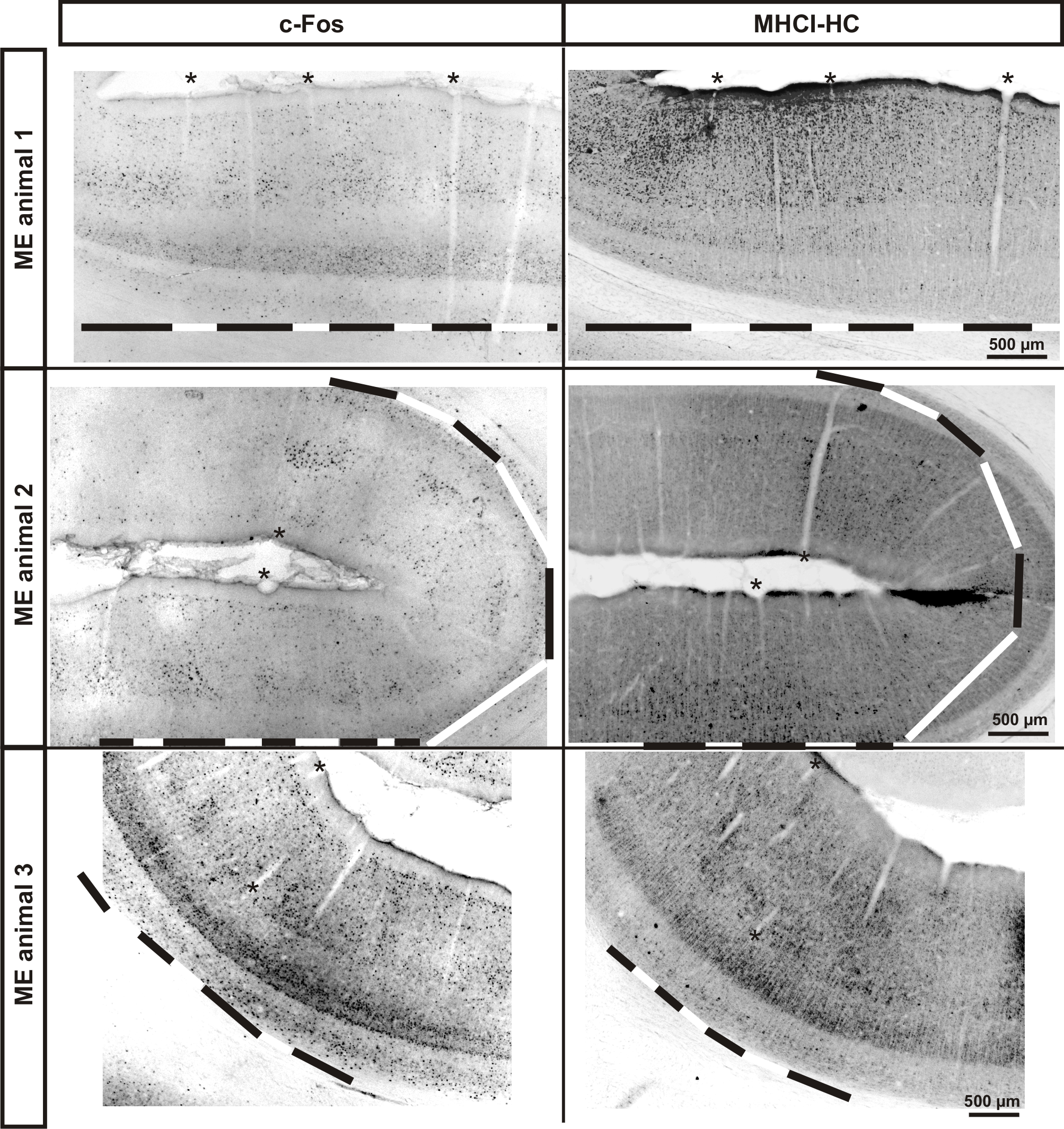

Supplement: Additional file 6 — Primary visual cortex of all enucleated animals reveals banded pattern of MHCI-HC and c-Fos immunoreactivity. Black horizontal lines in the lower parts of the images indicate areas with high MHCI-HC and c-Fos immunoreactivity; white lines point to low immunoreactivity areas. Animals ME1 and 2 are siblings. Asterisks denote identical blood vessels. Note: Middle panel, MHCI-HC staining: black region in the right part of the image is an artefact. Note: Black horizontal lines in upper panel in (B) indicate areas with high c-Fos immunoreactivity, while white lines point to low immunoreactivity areas. Scale bar: 0.5 mm. [file 1744-9081-7-1-S6.PNG]

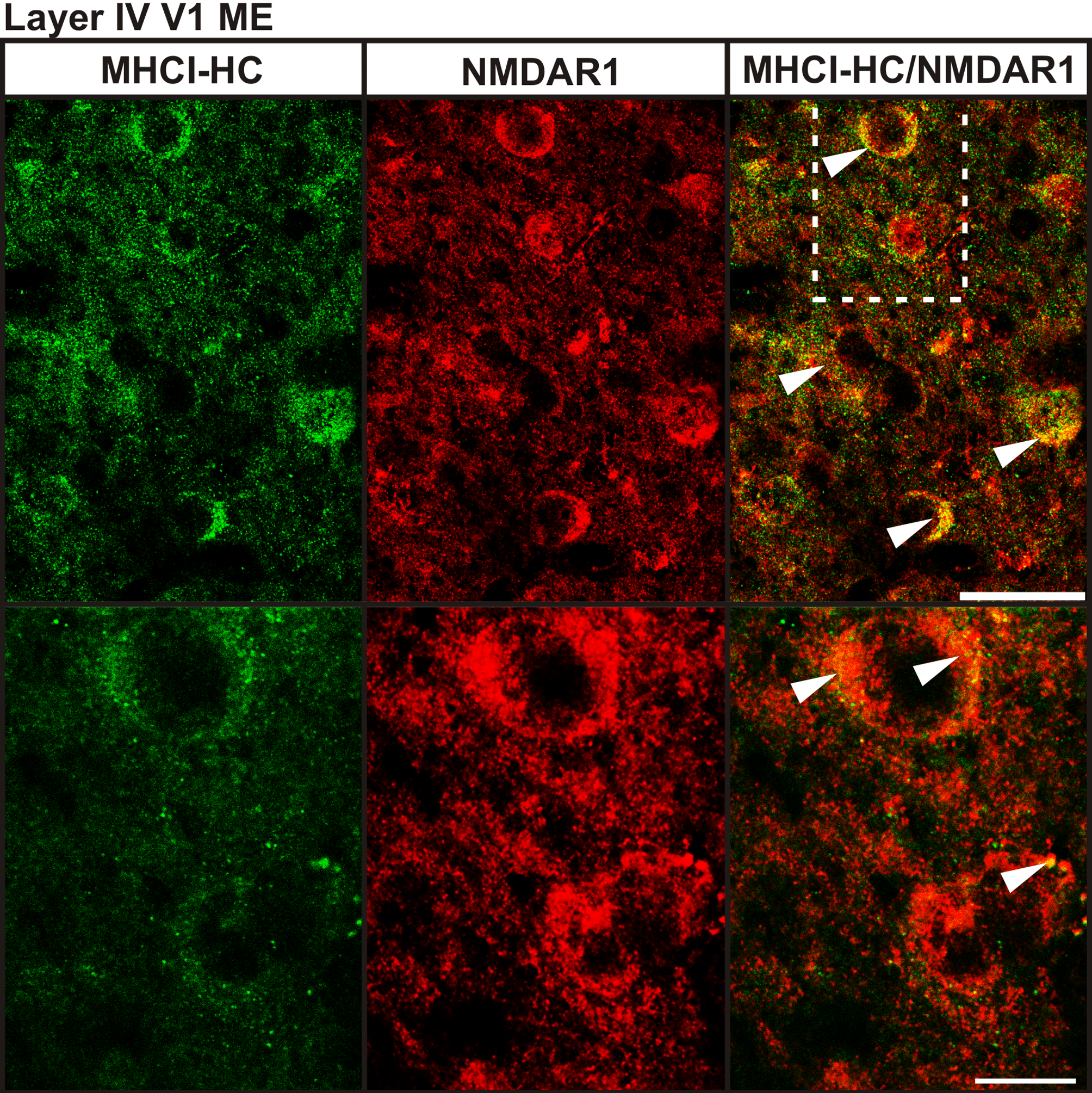

Supplement: Additional file 7 — MHCI-HC protein is localized on NMDAR1-positive neurons in the visual cortex of enucleated animals. MHCI-HC (A) is present on NMDAR1-positive neurons in the primary visual cortex of enucleated animals (B, white arrowheads in C). Higher magnification reveals MHCI-HC clusters (D) overlapping with NMDAR1 clusters (E, white arrowheads in F). Scale bar in C: 50 μm; scale bar in D: 25 μm. Abbreviations: ME, monocularly enucleated animals; V1, primary visual cortex. [file 1744-9081-7-1-S7.PNG]

## c-Fos

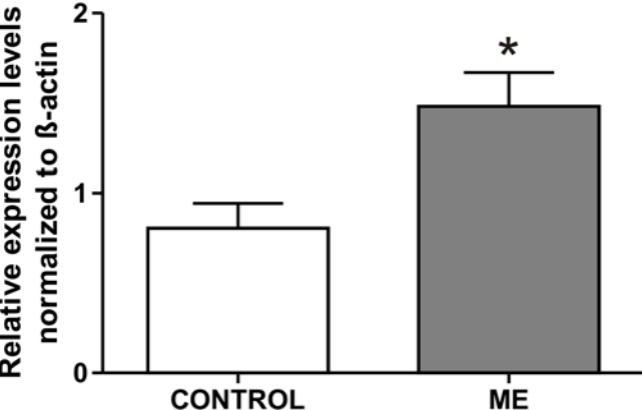

Supplement: Additional file 8 — c-Fos mRNA expression is upregulated in response to monocular enucleation. qRT-PCR reveals a significant difference in c-Fos mRNA expression levels in the whole visual cortices of animals that have undergone monocular enucleation (ME) and controls. Data are expressed as mean ± SEM (standard error of the mean) and are representative of three independent experiments performed with samples isolated from both hemispheres of N = 3 animals/group. Significant differences between groups as determined by Student's two-tailed t-test: *, p < 0.05 (t = 2.875 df = 10). [file 1744-9081-7-1-S8.PDF]

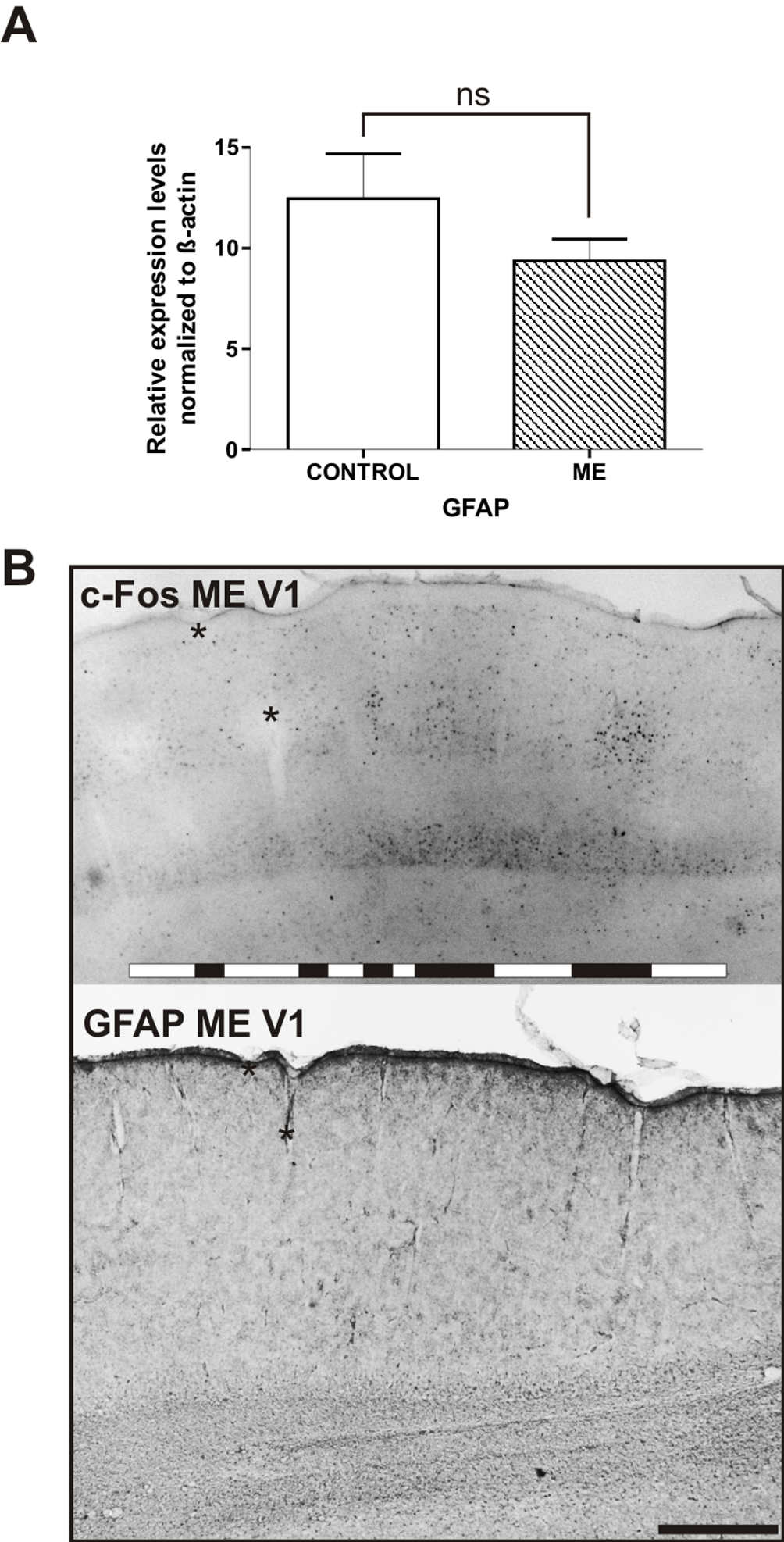

Supplement: Additional file 9 — Effects of monocular enucleation on the expression of GFAP mRNA and protein expression. A) Enucleation has no significant effect on the levels of GFAP mRNA in the visual cortex as determined with qRT-PCR. Abbreviations: ME, monocularly enucleated animals. Data are expressed as mean ± SEM (standard error of the mean) from 2 independent experiments, N = 3 animals/group (ns, not significant; Student's two-tailed t-test). B) Enucleation has no visible effect of GFAP immunoreactivity in the visual cortex of enucleated animals. Adjacent sections were processed for c-Fos and GFAP immunostaining. While c-Fos revealed patchy immunoreactivity in the visual cortex of enucleated animals (upper panel), GFAP revealed uniform staining pattern (lower panel). Abbreviations: ME, monocularly enucleated animals; V1, primary visual cortex. [file 1744-9081-7-1-S9.PNG]
