# Supplementary material for: Activity-dependent regulation of MHC class I expression in the developing primary visual cortex of the common marmoset monkey
Source: Behav Brain Funct. 2011 Jan 4;7:1. doi: 10.1186/1744-9081-7-1 (PMC3023691; doi:10.1186/1744-9081-7-1)
Supplement: Additional file 2 — Caja-G qRT-PCR yielded a single product corresponding to Caja-G locus only. After the qRT-PCR, the reaction product was purified using QIAquick PCR Purification Kit (QIAGEN) and sequenced using the forward primer used for the qRT-PCR reaction (bold and italics in the Caja-G sequence). The obtained sequence (bolded and highlighted in grey in the Caja-G sequence) was identified as Caja-G (alleles Caja-G*1, *3 and *5) using NCBI BLAST tool. Example of pairwise alignments generated by BLAST, as well as the BLAST hits table are shown in the lower part of the figure. [file 1744-9081-7-1-S2.PDF]

Callithrix jacchus MHC class I (Caja-G*01) mRNA, partial cds
GenBank: U59637.1

>gi|1389920|gb|U59637.1|CJU59637 Callithrix jacchus MHC class I (Caja-G*01) mRNA, partial cds

CTCCTCCTGTTGCTCTCGGGGGCCTTGGTCCTGACTGAGACCTGGGCAGGCTCCCACTCCATGAGGTATT
TCTACACCTCCGTGTCCCGGCCCGGCCGCGGGGAGCCCCGCTTCATCATCGTGGGCTACGTGGACGACAC
GCAGTTCGTGCGGTTCGACAGCGACGCCGCGAATCCGAGGATGGAGCCGCGGGCGCCGTGGGTGGAGCAG
GAGGGGCCGGAGTATTGGGAGGAGCAGACACGGAGAGCCAAGGCCCACGCACAGACTGACCGAGTGGACC
TGCGGACCCTCCGCGGCTACTACAACCAGAGCGACGCGGGGTCTCACACCATCCAGTGGATGTCTGGCTG
CGTAGTGGGGCCGGACGGGCGCCTCCTCCGTGGGTACCGGCAGGACGCCTACGACGGCAAGGACTACATC
GCCCTGAACGAGGACCTGCGCTCCTGGACGGCCGCGGACATGGCGGCTCAGATCACCCAGCGCAAGTGGG
AGGCGGCCAATGCGGCTGAGGAGATGAGAGCCTACCTGGAGGGCGAGTGCCTGGAGTGGCTCCACAGATA
CCTGGAGAACGCGAAGGAGACGCTGCAGCGAGCGGAGCCCCCTAAGACACACGTGACCCACCACCCCGTC
TCTGACCATGAGGCCACCCTGAGGTGCTGGGCCCTGGGCTTCTACCCTGCGGAGATCACACTGACCTGGC
AGCGGGATGGGGAGGACCAGACCCAGGACATGGAGCTCGTAGAGACCAGGCCCACAGGGGATAGAACCTT
CCAGAAGTGGGCTGCTGTGGTGGTGCCTTCTGGAGAGGAGCACAGATACACATGCCATGTGCAGCACGAG
GGGCTGCCCGAGCCCCTCACCCTGAGATGGGAGCCGCCTTCCCAGCCCACCATCCCCATCATGGGCATCG
TGGCTGCCTTGGCTATCCTTGGAGCAGTCGGTGGAGCTGTGGTCGTTGCT***GTGATGTGGAGGAAGAAGAG***
CTCAGACAAAAAAGG**AGGAAGCTACTCTCAGGCTGCAAGAAGCGAGAGTGCCCAGGGCTCTGATGTGTCT**
CTCACGGCTTGTAAAGTGTGA

```
>gb|U59641.1|CJU59641 Callithrix jaccus MHC class I (Caja-G*05) mRNA, partial cds
Length=1071

 Score = 67.9 bits (34),  Expect = 3e-09
 Identities = 41/42 (98%), Gaps = 1/42 (2%)
 Strand=Plus/Plus

Query  8     GGCTGCAAGA-GCGAGAGTGCCCAGGGCTCTGATGTGTCTCT  48
             |||||||||| |||||||||||||||||||||||||||||||
Sbjct  1011  GGCTGCAAGAAGCGAGAGTGCCCAGGGCTCTGATGTGTCTCT  1052


>gb|U59639.1|CJU59639 Callithrix jaccus MHC class I (Caja-G*03) mRNA, partial cds
Length=1071

 Score = 67.9 bits (34),  Expect = 3e-09
 Identities = 41/42 (98%), Gaps = 1/42 (2%)
 Strand=Plus/Plus

Query  8     GGCTGCAAGA-GCGAGAGTGCCCAGGGCTCTGATGTGTCTCT  48
             |||||||||| |||||||||||||||||||||||||||||||
Sbjct  1011  GGCTGCAAGAAGCGAGAGTGCCCAGGGCTCTGATGTGTCTCT  1052


>gb|U59637.1|CJU59637 Callithrix jacchus MHC class I (Caja-G*01) mRNA, partial cds
Length=1071

 Score = 67.9 bits (34),  Expect = 3e-09
 Identities = 41/42 (98%), Gaps = 1/42 (2%)
 Strand=Plus/Plus

Query  8     GGCTGCAAGA-GCGAGAGTGCCCAGGGCTCTGATGTGTCTCT  48
             |||||||||| |||||||||||||||||||||||||||||||
Sbjct  1011  GGCTGCAAGAAGCGAGAGTGCCCAGGGCTCTGATGTGTCTCT  1052
```

Sequences producing significant alignments:

Accession	Description	Max score	Total score	Query coverage	E value	Max ident	Links
U59641.1	Callithrix jaccus MHC class I (Caja-G*05) mRNA, partial cds	67.9	67.9	85%	3e-09	97%	
U59639.1	Callithrix jaccus MHC class I (Caja-G*03) mRNA, partial cds	67.9	67.9	85%	3e-09	97%	
U59637.1	Callithrix jacchus MHC class I (Caja-G*01) mRNA, partial cds	67.9	67.9	85%	3e-09	97%	
XR_091099.1	PREDICTED: Callithrix jacchus class I histocompatibility antigen, A alpha chain-like (LOC100401501), miscRNA	60.0	60.0	85%	8e-07	95%	G M
EU403493.1	Macaca mulatta MHC class I antigen (Mamu-A) mRNA, Mamu-A1*0410 allele, partial cds	60.0	60.0	85%	8e-07	95%	U
